# The Aquaporin‐4 Expression and Localization in the Olfactory Epithelium Modulate the Odorant‐Evoked Responses and Olfactory‐Driven Behavior

**DOI:** 10.1002/glia.70024

**Published:** 2025-04-18

**Authors:** Donatella Lobraico, Pasqua Abbrescia, Maria Grazia Fioriello, Barbara Barile, Claudia Palazzo, Onofrio Valente, Grazia Paola Nicchia, Michele Dibattista, Antonio Frigeri

**Affiliations:** ^1^ Department of Translational Biomedicine and Neuroscience (DiBraiN), School of Medicine University of Bari Aldo Moro Bari Italy; ^2^ Department of Biosciences, Biotechnology and Environment University of Bari Aldo Moro Bari Italy

**Keywords:** AQP4 isoforms, AQP4ex, AQP4M23, Aquaporin‐4, olfactory epithelium, sustentacular cells

## Abstract

Aquaporin‐4 (AQP4) is a water‐selective channel expressed in glial cells throughout the central nervous system (CNS). It serves as the primary water channel in the neuropil and plays roles in physiological functions, including regulating water homeostasis by adjusting cell volume and modulating neuronal activity. Different isoforms of AQP4 are expressed in glial‐like cells known as sustentacular cells (SUSs) of the olfactory epithelium (OE). Notably, mice lacking all AQP4 isoforms exhibit impaired olfactory abilities. Therefore, we aim to uncover the physiological role of AQP4 isoforms, particularly the AQP4ex isoforms (AQP4M1ex, AQP4M23ex) and the orthogonal array of particles (OAPs)‐forming isoform (AQP4M23) in the OE. We investigated the impact of AQP4 isoforms on the OE, observing a reduced number of mature olfactory sensory neurons (OSNs), SUSs, and globose basal cells (GBCs) in mice lacking AQP4ex (AQP4ex‐KO) or OAPs (OAP‐null). This suggests that AQP4 isoforms are involved in maintaining an optimal microenvironment in the OE, preserving cell density. Next, we explored the role of AQP4 in modulating odorant‐evoked responses through electro‐olfactogram recordings, where we found reduced odorant responses in mice lacking AQP4 isoforms. Assessments of olfactory ability revealed deficits in odor‐guided food‐seeking behavior in both AQP4ex‐KO and OAP‐null mice. Furthermore, AQP4ex‐KO mice exhibited a diminished ability to discriminate between different odorants, while OAP‐null mice were unable to recognize them as distinct. Overall, our data highlight the role of AQP4 isoforms in modulating neuronal homeostasis, influencing odorant‐evoked responses and cell density in the OE, with AQP4ex emerging as a key regulator despite its low abundance.

## Main Points

- AQP4ex and OAP‐forming AQP4M23 are expressed in non‐neuronal cells of the olfactory epithelium.
- AQP4ex‐KO and OAP‐null mice show reduced cell density, including OSNs, and impaired odor responses and behavior, with AQP4ex emerging as key regulator.

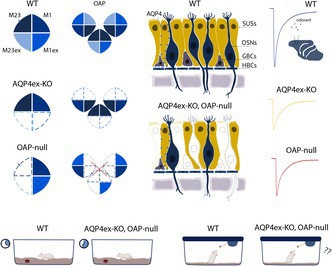

## 1 Introduction

Olfaction is one of the most ancient sensory systems, and its functional unit, the olfactory sensory neuron (OSN) is located in the olfactory epithelium (OE) (Buck and Axel 1991; Niimura 2012; Arguello et al. 2021). Since the initial description of the OE, cell types other than OSNs, such as sustentacular cells (SUSs), have been identified (Allison 1953). SUSs enwrap mature OSNs, suggesting that they provide mechanical, trophic, and metabolic support while also modulating the electrical activity of OSNs (Morrison and Costanzo 1992; Nomura et al. 2004; Liang 2018; Hernandez‐Clavijo et al. 2021). Indeed, SUSs influence OSN activity by producing a variety of neuromodulatory molecules, including NPY, an anorexigenic peptide, BDNF (brain‐derived neurotrophic factor), and ATP, among others, which can modulate the odorant‐evoked electrical response in OSNs by increasing Ca^2+^ influx (Hansel et al. 2001; Hayoz et al. 2012; Frontera et al. 2015; Henriques et al. 2019). Various modulatory activities promoted by SUSs are associated with nucleotide signaling in the OE. SUSs express metabotropic P2Y purinergic receptors, and ATP induces intracellular Ca^2+^ influx in both SUSs and OSNs, thus modulating the odorant‐evoked electrical response in OSNs (Hegg et al. 2003, 2009; Dooley et al. 2011). SUSs can sustain OSN activity by releasing glucose into the mucus that covers the OE. This glucose is then taken up into OSN cilia and metabolized to generate ATP, which sustains olfactory transduction (Villar et al. 2017; Acevedo et al. 2019). Modulatory activity may also be mediated by gap junctions, providing cytoplasmic continuity and electrical coupling between SUSs, which leads to intercellular communication via Ca^2+^ and other signaling molecules (Rash et al. 2005; Vogalis et al. 2005a, 2005b; Hegg et al. 2009). Despite their differing embryological origins, the involvement of SUSs in neuroexcitatory and/or neuromodulatory processes makes them functionally similar to astrocytes. These cells adjust their activity to support and synchronize local electrical signals, thereby influencing synaptic plasticity (Parpura et al. 1994; Duan et al. 2003; Nedergaard et al. 2003; Jourdain et al. 2007; Rouach et al. 2008). Such plasticity also relies on the water channel aquaporin‐4 (AQP4) (Kitaura et al. 2009; Skucas et al. 2011; Li et al. 2012; Scharfman and Binder 2013; Ciappelloni et al. 2019).

AQP4 is a water channel primarily expressed in astrocyte end‐feet and ependymal cells surrounding the ventricles (Frigeri et al. 1995; Nielsen, Nagelhus, et al. 1997; Rash et al. 1998). AQP4 plays a significant role in facilitating water movements in the central nervous system (CNS) and is crucial for regulating blood–brain barrier (BBB) water permeability (Nicchia et al. 2004; Oshio et al. 2004; Papadopoulos et al. 2004). The water channel has two main isoforms: a longer isoform, starting with Met‐1, called AQP4M1 (32 kDa), and a shorter isoform with a translation starting point at Met‐23, known as AQP4M23 (30 kDa), which is produced by an alternative splicing mechanism called leaky scanning (Rossi et al. 2010). AQP4 may also undergo an alternative translational modification known as translational readthrough, resulting in an extension of approximately 29 amino acids at the C‐terminus, thus generating the extended isoforms (AQP4ex) of either AQP4M23 (AQP4M23ex) or AQP4M1 (AQP4M1ex) (Loughran et al. 2014; De Bellis et al. 2017). Unlike other AQPs, which assemble into homotetramers and form intramembrane particles, the AQP4M1 and AQP4M23 isoforms are assembled in cell membranes as AQP4 heterotetramers (Lu et al. 1996; Furman et al. 2003). Freeze‐fracture electron microscopy has revealed square arrays of intramembrane particles in cell membranes expressing AQP4, termed orthogonal array of particles (OAPs) (Landis and Reese 1974; Crane et al. 2009; Hirt et al. 2011; Jin et al. 2011). The ratio of AQP4M1 and AQP4M23 expression influences the size of the OAPs (Silberstein et al. 2004; Nicchia et al. 2010; Rossi et al. 2010). Furthermore, AQP4ex is essential for anchoring OAPs at the perivascular astrocyte end‐feet, and its absence leads to rearrangement and redistribution of OAPs (Palazzo et al. 2019, 2020; Pati et al. 2022).

AQP4 expression has been observed in the mouse OE, particularly in the basal cells (BCs), Bowman's glands, and SUSs (Nielsen, King, et al. 1997; Ablimit et al. 2006; Solbu and Holen 2012). Despite this, our understanding of AQP4's function is limited, especially regarding the AQP4ex isoforms in peripheral sensory organs like the OE. Conversely, AQP4's role in maintaining an optimal microenvironment in the extracellular space (ECS) is well established. This raises several important questions: could AQP4 serve similar functions in the SUSs of the OE? What role do AQP4 isoforms play? To investigate these aspects, we used AQP4ex‐KO mice, deficient in AQP4M1ex and AQP4M23ex isoforms, alongside the OAP‐null mouse model, which lacks AQP4M23 and AQP4M23ex isoforms. Through this approach, we examined the contribution of OAPs in the OE, and for the first time reported the localization of the newly identified AQP4ex isoforms in the OE, exploring their role in olfactory physiology and behavior.

## 2 Results

### 2.1 Localization of AQP4 and AQP4ex in the Olfactory Epithelium

The main water channel of the nervous system, AQP4, has different isoforms that have been described (Hasegawa et al. 1994; Jung et al. 1994; De Bellis et al. 2017). The extended isoforms produced via translational readthrough have been shown to be crucial for the proper cellular compartmentalization of the water channel in glial cells of different districts in the brain (De Bellis et al. 2017; Palazzo et al. 2019, 2020). It remains unclear whether the extended isoforms are expressed in the sensory organs and their functional roles.

**FIGURE 1:**
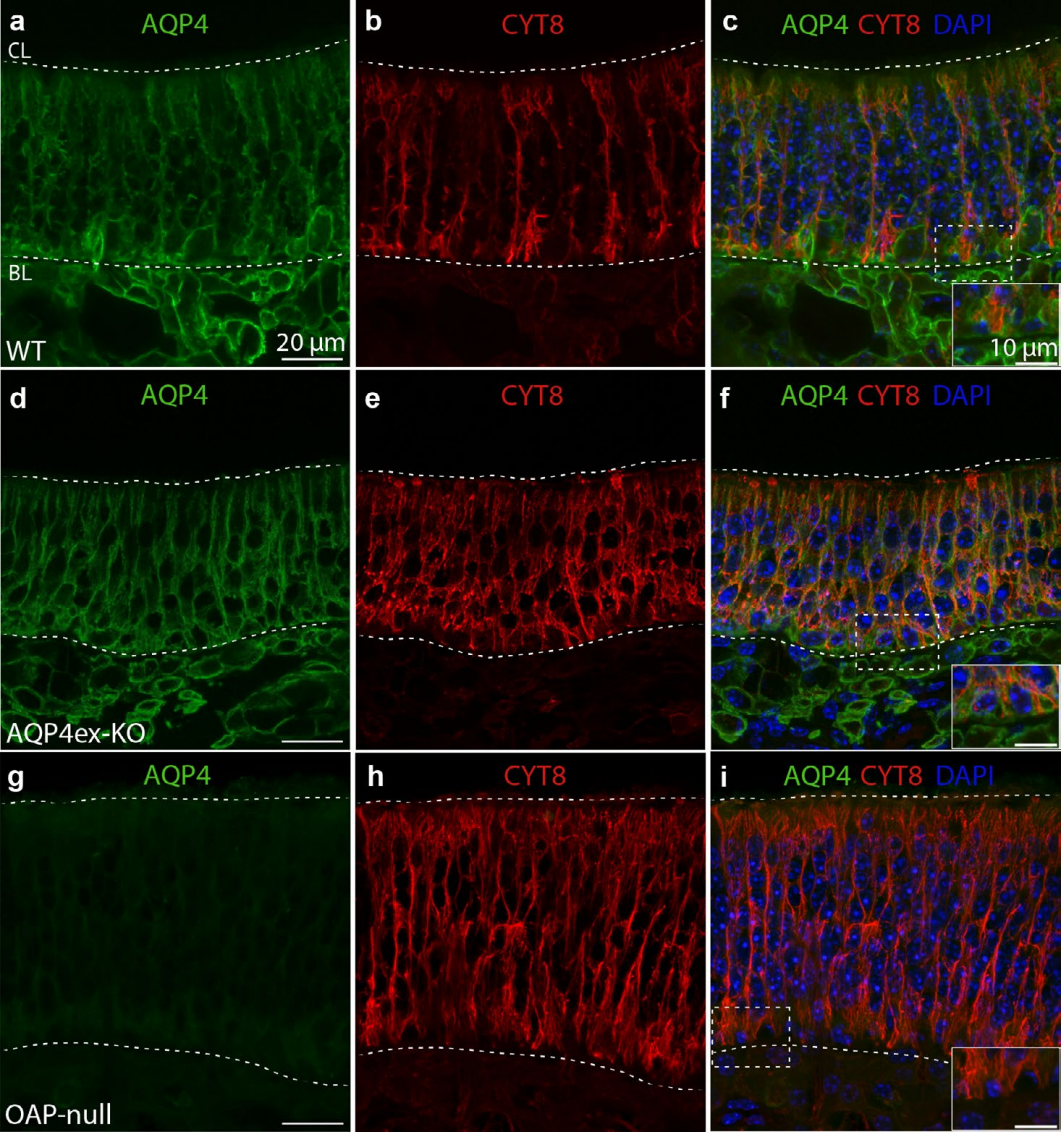
AQP4 is expressed on the basolateral membrane of the sustentacular cells. (a, d, g) High‐magnification confocal micrographs of coronal sections from WT, AQP4ex‐KO, and OAP‐null mouse olfactory epithelium showing AQP4 expression pattern. The ciliary layer (CL) and the basal lamina (BL) are shown. (b, e, h) Cytokeratin 8 (CYT8) was used to stain the SUSs. (c, f, i) AQP4 staining is confined to the basolateral membrane of the SUSs (inset showing the basal staining of AQP4 and CYT8, surrounding the basal cells). AQP4 is also localized beneath the basal lamina (BL). (g, i) A sharp decrease in the staining is detectable in OAP‐null.

**FIGURE 2:**
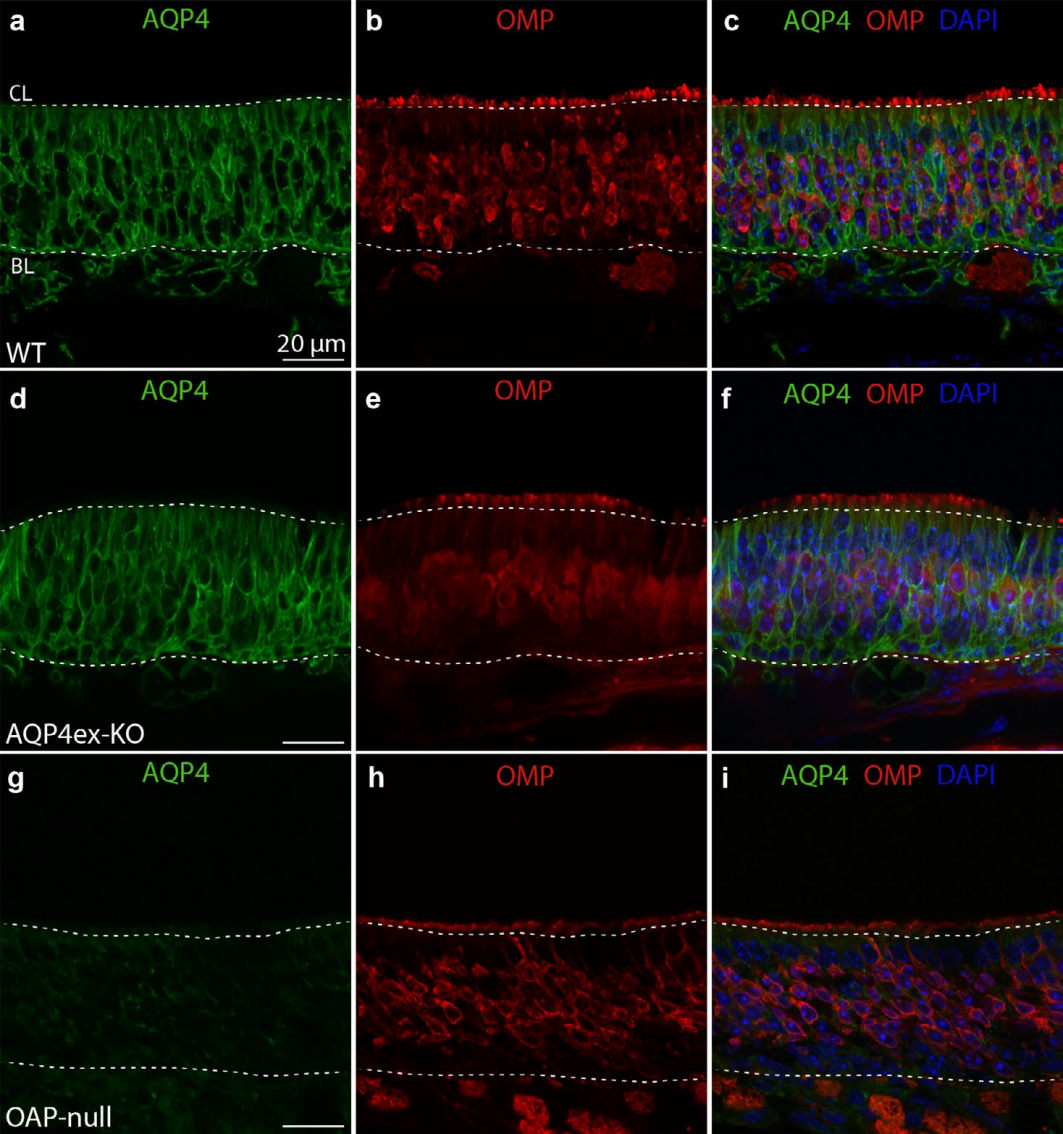
AQP4 is not expressed in the OSNs. (a, d, g) Confocal micrographs of coronal sections from WT, AQP4ex‐KO, and OAP‐null mouse olfactory epithelium showing AQP4 expression pattern. (b, e, h) OMP was used to stain the OSNs. (c, f, i) AQP4 is not localized in the OSNs. AQP4 staining also surrounds the nuclei of the cells relying on the basal lamina. (g, i) A sharp decrease in the staining is detectable in OAP‐null.

Using an antibody that detects all AQP4 isoforms, along with cytokeratin 8 (CYT8) to stain SUSs (Maurya et al. 2015), we identified AQP4 localization on the basolateral membrane of SUSs (Figure 1c–i). Additionally, AQP4 encases the BCs laying on the basal lamina (BL) (Figure 1c–i inset). AQP4 staining appears more extensive than CYT8 labeling, suggesting that AQP4 is not only present in the basal processes of SUSs but also in the BCs (Figure 1c–i inset; Figure 2c–i). We also discovered that AQP4 is expressed in the cells beneath the BL, most likely olfactory ensheathing cells (OECs) (Figures 1a,d and 6e). We did not observe any alteration in the AQP4 staining pattern in AQP4ex‐KO (Figure 1d–f). Finally, the weak and nearly absent staining of AQP4 in OAP‐null mice (Figure 1g–i) indicates that AQP4M23 isoform‐dependent OAP assembly is crucial for maintaining normal AQP4 expression levels in the OE, as demonstrated in the brain (De Bellis et al. 2021).

**FIGURE 3:**
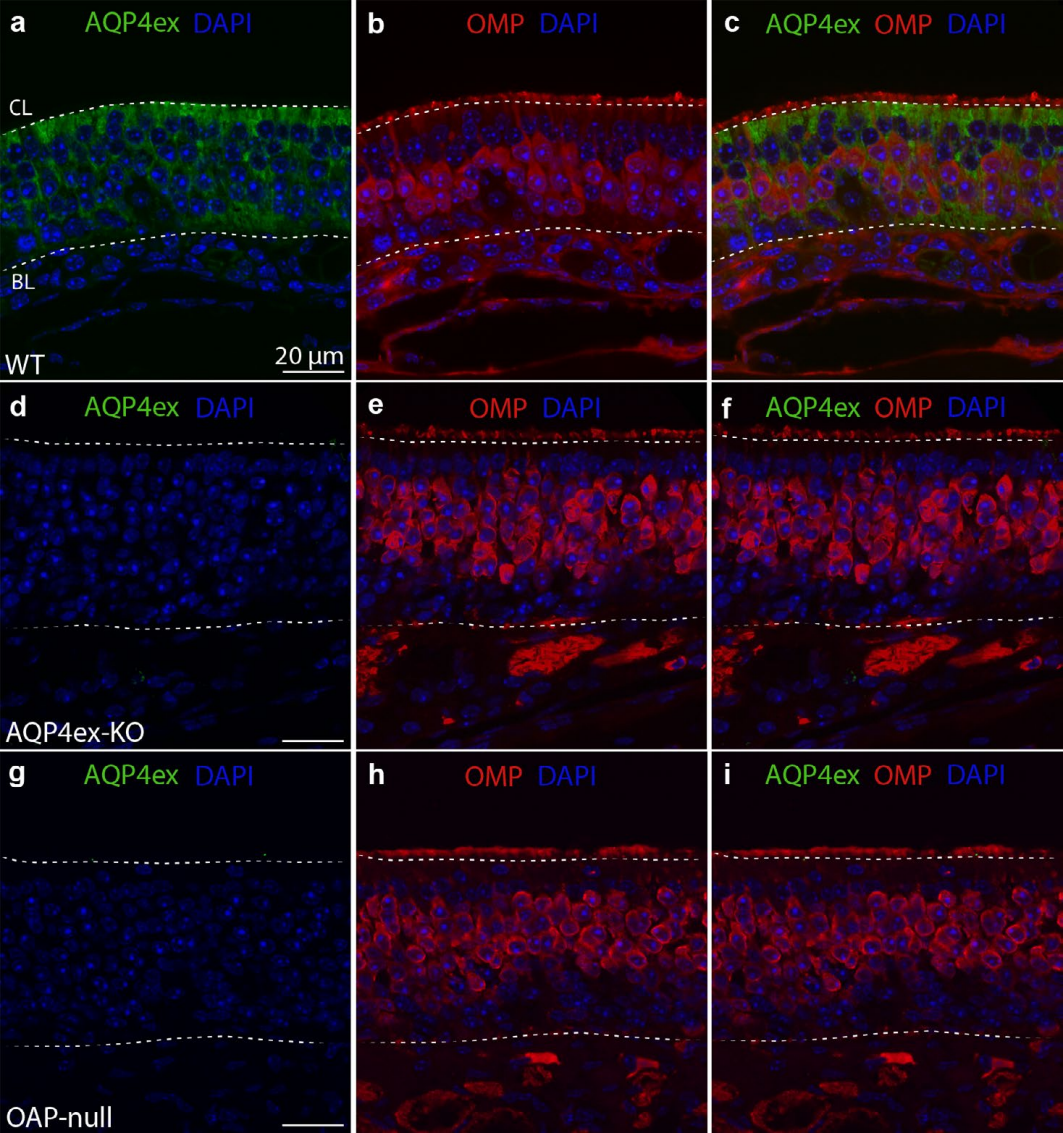
AQP4ex is localized on the SUSs. (a, d, g) Confocal micrographs of coronal sections from WT, AQP4ex‐KO, and OAP‐null mouse olfactory epithelium show AQP4ex expression patterns. (a–c) AQP4ex is localized on the SUSs, since it wraps the OSNs similarly to AQP4 staining. (d–f) AQP4ex staining is totally abolished in AQP4ex‐KO; (g–i) and it is almost absent in OAP‐null, hence the staining comes from AQP4M1ex isoform, which is almost totally absent.

Since AQP4 is expressed on the basolateral membrane of the SUSs, it wraps around the membrane of OSNs in the OE, where the OSN cell bodies are located. Furthermore, AQP4 expression is mutually exclusive with olfactory marker protein (OMP) staining, which is a marker for mature OSNs (Figure 2c–i). By using an antibody that specifically detects the extended isoforms of AQP4 (AQP4ex, see Methods), we observed that AQP4ex isoforms are also present in the OE, and they are not expressed in the OSNs. The staining pattern of these isoforms appears somewhat more diffuse in the cells compared to that of AQP4 staining (Figure 3a–c). Additionally, AQP4ex isoforms seem localized in the BCs, as the staining is also detectable at the bottom of the OE, although we cannot completely rule out the possibility that the signal could resemble the compartmentalization of AQP4ex in the basal feet of the SUSs (Figure 3a–c). Finally, the AQP4ex staining is barely detectable in OAP‐null, indicating that AQP4M1ex is very poorly expressed (Figure 3g–i).

**FIGURE 4:**
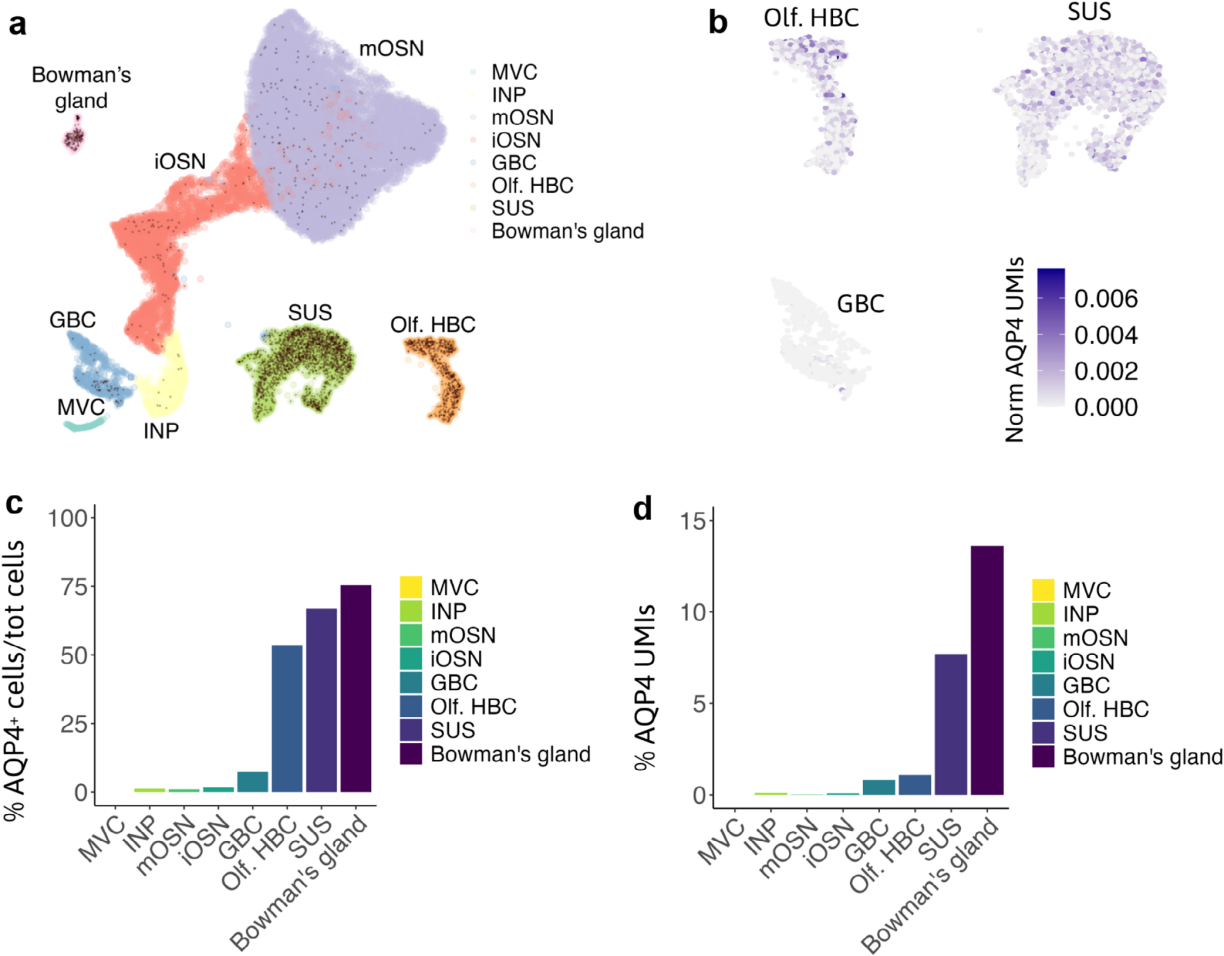
mRNA AQP4 expression in the OE cell types. (a) UMAP representation of scRNA‐seq data from the entire OE, colored by cell type (GBC: globose basal cells, INP: immature neuronal progenitors, iOSN: immature OSN, mOSN: mature OSN, MVC: microvillar cells, Olf. HBC: olfactory horizontal basal cells, SUS: sustentacular cells). AQP4^+^ cells are represented as colored dots. (b) Normalized AQP4 expression in each cell type. The number of transcripts (UMIs) for AQP4 was divided by the total number of UMIs for each cell. (c) Histograms illustrating the mean percentage of AQP4^+^ cells in each cell type, divided by the total number of cells of that type. (d) Histograms showing the mean percentage of AQP4 UMIs in each cell type. scRNA‐seq data produced by Brann et al. (2020).

To assess the AQP4 expression levels in the different cell types populating the OE, we investigated the transcriptional profile of AQP4 by analyzing previously published scRNA‐seq data from the OE by Brann et al. 2020. We found that AQP4 is predominantly expressed in the Bowman's gland, SUSs, olfactory HBCs, and globose basal cells (GBCs) (Figure 4a). AQP4 expression varies among these cells, with a significant number of Bowman's gland cells, SUSs, and olfactory HBCs expressing AQP4 (Figure 4b–d).

### 2.2 AQP4ex and AQP4M23 Contribute to the Structural Integrity of the Olfactory Epithelium

**FIGURE 5:**
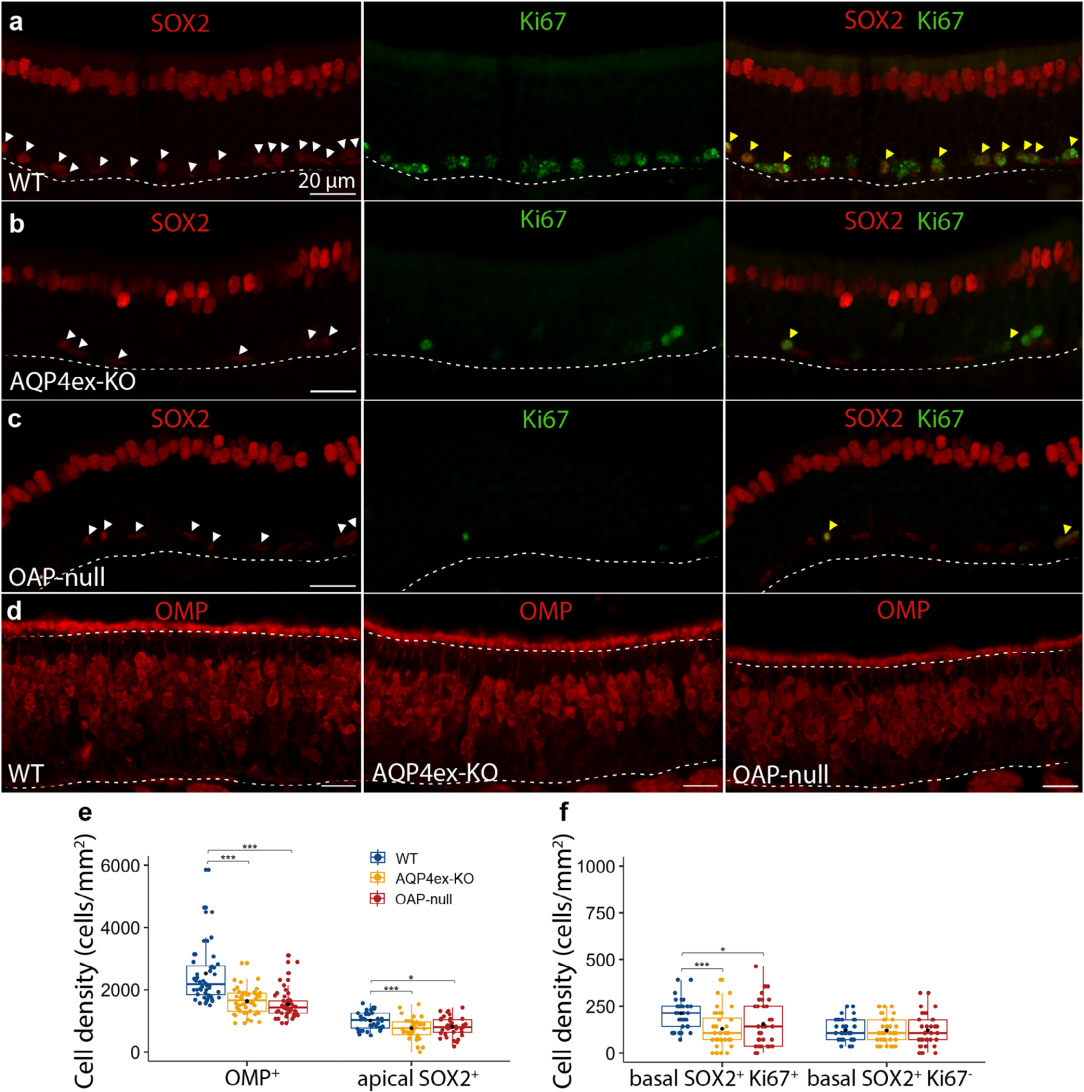
AQP4ex and AQP4M23 deletion affect cell density in the olfactory epithelium. (a, b, c) Coronal sections from WT, AQP4ex‐KO and OAP‐null olfactory epithelium showing SUSs nuclei and basal cells stained with Sox2, and proliferative GBCs stained with Ki67. White arrowheads in the pictures showing SOX2 staining (left), represent all the SOX2^+^ cells relying on the basal lamina (dashed line). Yellow arrowheads in the pictures showing SOX2 and Ki67 staining (right) represent the SOX2^+^‐Ki67^+^ cells. (d) Coronal sections from WT, AQP4ex‐KO and OAP‐null olfactory epithelium showing OMP^+^ cells. (e, f) Box plots showing cell density in WT and KO mice. The central point represents the mean, central line: Median, upper and lower box boundaries: 25th and 75th percentile, extreme lines: The highest and lowest value. SUSs were counted considering the apical SOX2^+^ cells. GBCs were counted as basal SOX2^+^‐Ki67^+^. Other basal SOX2^+^‐Ki67^−^ were counted, and they represent quiescent GBCs and/or HBCs. One‐way ANOVA, followed by Tukey test post hoc analysis, or Kruskal–Wallis test followed by Benjamini–Hochberg (BH) post hoc analysis was performed. Anova I performed on apical SOX2^+^ (genotype: *F*_(2,105)_ = 6.21, *p* = 3e^−3^). Kruskal–Wallis test was applied to OMP^+^: *H*_(2)_ = 52.71, *p* = 3.58e^−12^. Kruskal–Wallis test was conducted on basal SOX2^+^ Ki67^+^: *H*_(2)_ = 13.39, *p* = 1.24e^−3^; *p* = *0.05, **0.01, ***0.001; *n* = 3 for each genotype.

The nuclei of SUSs and BCs were identified using an antibody against the transcription factor Sox2 (Guo et al. 2010). The nuclei of SUSs are located in the apical part of the OE, while the nuclei of BCs are found in the basal part of the OE, above the BL (Figure 5a–c).

While performing immunofluorescence experiments, we observed that the cell density in the OE of AQP4ex‐KO and OAP‐null models appeared sparser and lower than WT. Consequently, we conducted cell counts and discovered that mature OSNs, stained with OMP, were approximately 30% fewer in the KO models compared to WT (WT: 2524.55 ± 151.28 OMP^+^ cells/mm^2^ (mean cell density ± SE), AQP4ex‐KO: 1631.69 ± 59.14, OAP‐null: 1529.76 ± 66.07) (Figure 5d,e). Additionally, the SUSs counted as apical SOX2^+^ nuclei in the apical part of the OE were significantly fewer in the KO models than in WT (WT: 1008.93 ± 46.62 apical SOX2^+^ cells/mm^2^, AQP4ex‐KO: 765.873 ± 56.10, OAP‐null: 818.45 ± 50.84) (Figure 5a–c,e).

SOX2 is also a marker for basal stem cells in the OE (Fukuyama et al. 2015). This cell population is quite heterogeneous, consisting of both HBCs and GBCs; therefore, we used Ki67, a marker for proliferating cells (Guo et al. 2010; Jang et al. 2014), and categorized SOX2^+^‐Ki67^+^ cells as proliferative GBCs. The population of BCs that are positive for both SOX2 and Ki67 was significantly lower in AQP4ex‐KO and OAP‐null compared to WT (WT: 213.29 ± 13.98 SOX2^+^‐Ki67^+^ cells/mm^2^, AQP4ex‐KO: 129.96 ± 16.29, OAP‐null: 154.76 ± 20.96) (Figure 5a–c,e). Furthermore, we identified a population of SOX2^+^‐Ki67^−^, most of which formed a flat monolayer on the BL consistent with the HBCs phenotype (Guo et al. 2010); however, we could not rule out the possibility that some quiescent GBCs were also among them. The density of this cell population did not differ across the genotypes (WT: 123.01 ± 9.90 SOX2^+^‐Ki67^−^ cells/mm^2^, AQP4ex‐KO: 121.03 ± 11.76, OAP‐null: 122.02 ± 14.33) (Figure 5a–c,e).

**FIGURE 6:**
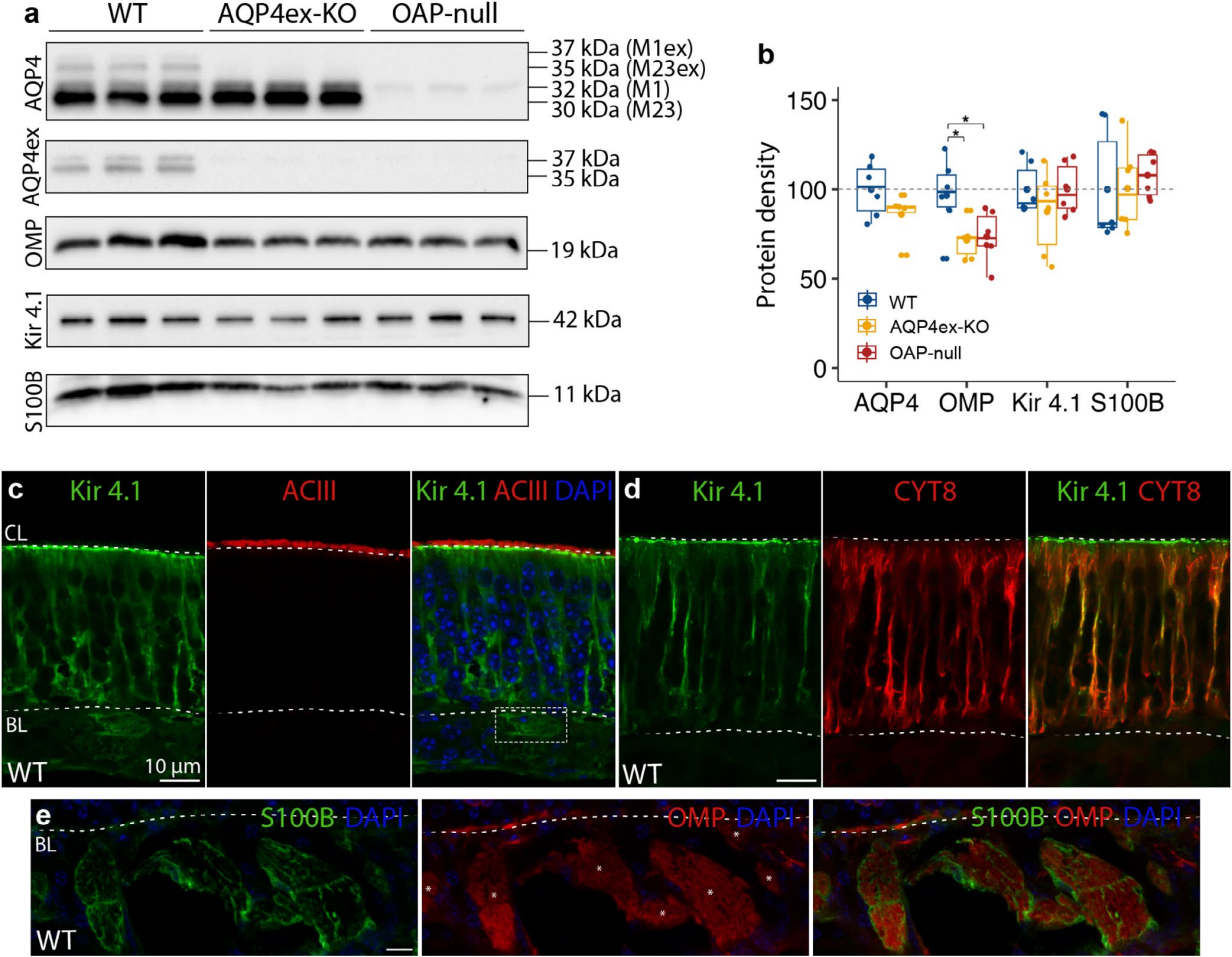
OMP expression is reduced in AQP4ex‐KO and OAP‐null. (a) Western blot was performed on olfactory epithelium proteins: AQP4, AQP4ex, OMP, Kir 4.1, and S100B. Membranes probed with anti‐AQP4 antibody, showing proteins at 30, 32, 35, 37 kDa corresponding to the four AQP4 isoforms. (b) Box plots showing protein density of AQP4, OMP, Kir 4.1, and S100B in WT and KO mice. The central point represents the mean, central line: Median, upper and lower box boundaries: 25th and 75th percentile, extreme lines: The highest and lowest value. One‐way Anova followed by Tukey test post hoc analysis was conducted. In each lane, the intensity of the analyzed band was first normalized to the total protein loaded, visualized by Ponceau staining. Hence, values from all samples were expressed relative to the mean of the WT group and reported as a percentage. Anova I performed on OMP density revealed genotype as the main effect (genotype: *F*_(2,15)_ = 4.66, *p* = 2.7e^−2^; *p* = *0.05, **0.01, ***0.001; *n* = 6 per genotype). (c) Confocal micrographs of coronal sections from WT mouse olfactory epithelium showing Kir 4.1 and ACIII (marker of ciliary layer) staining. The ciliary layer and basal lamina (BL) are shown (dashed lines). Kir 4.1 is localized on the SUSs membrane and their microvilli. Kir 4.1 staining is also present beneath the basal lamina, probably expressed by the olfactory ensheathing cells (OECs). (d) Kir 4.1 and CYT8 staining are shown. (e) S100B (marker of the OECs) staining is shown beneath the basal lamina (dashed line). OMP was used to stain the axon bundles (asterisks).

All these findings were further corroborated by western blot analysis. Here, we observed that the density of OMP protein relative to the total protein quantity is decreased in the KO models compared to WT (Figure 6a,b). Moreover, we could distinguish the bands corresponding to different AQP4 isoforms, indicating that AQP4ex is less abundant in the OE compared to the overall AQP4, while the AQP4M23 isoform is more abundant than AQP4M1 (Figure 6a). Densitometric analysis indicates that AQP4ex constitutes roughly 6% of the total AQP4 in the OE of WT mice. Moreover, the absence of AQP4ex isoforms does not significantly affect the quantities of the other AQP4 isoforms (Figure 6b).

In both KO mouse models, we did not observe any altered expression of Kir 4.1 (Figure 6a,b), an inward‐rectifying potassium channel expressed in SUSs, as demonstrated by its colocalization with CYT8 (Figure 6d). Kir 4.1 is primarily localized in the microvilli of the SUSs, with its staining appearing just beneath ACIII, a protein involved in olfactory transduction expressed in OSNs' cilia (Jones and Reed 1989; Maurya et al. 2015; Agostinelli et al. 2021; Figure 6c).

Furthermore, we found that Kir 4.1 is localized beneath the BL (Figure 6c). The structures marked with Kir 4.1 resemble OECs. Indeed, it has been reported that Kir4.1 is expressed by OECs in the BL propria of the mouse OE (Smith et al. 2020). When using S100B, a marker for OECs (Afhami Mina et al. 2018; Smith et al. 2020), we observed a similar staining pattern, albeit more intense, and it does not colocalize with OMP (Figure 6e). Although AQP4 is abundantly expressed in OECs, these cells do not show changes in density, as quantified by S100B through western blot analysis (Figure 6a,b), and we found no differences between the WT and KO mouse models. In summary, our results indicate that AQP4ex expression and the OAP‐forming isoform AQP4M23 are necessary to maintain the proper ratio between the different cell types in the OE, suggesting that they contribute to establishing a permissive microenvironment for cell development and differentiation.

### 2.3 EOG Responses Are Reduced in AQP4ex‐KO and OAP‐Null Mouse Model

**FIGURE 7:**
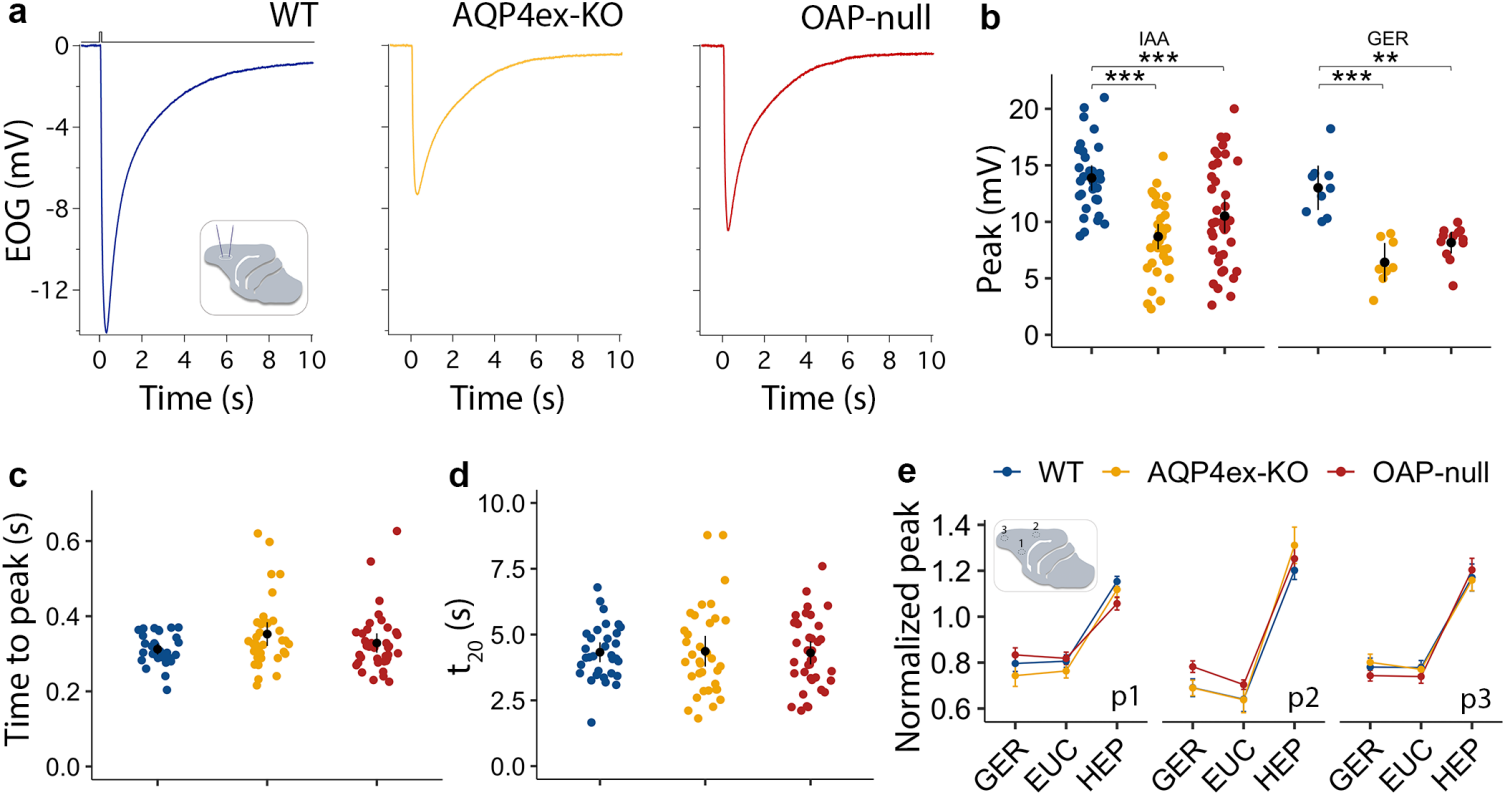
AQP4 isoforms affect the odorant‐evoked response. (a) Odorant‐evoked EOG responses to 100 ms exposure to isoamyl acetate (10^−1^ M) were recorded from turbinate IIa of WT (blue), AQP4ex‐KO (yellow) and OAP‐null (red). (b) EOG amplitude response to 10^−1^ M isoamyl acetate and geraniol. EOG responses are reduced in AQP4ex‐KO and OAP‐null mice; data are presented as mean and ci. Two‐way Anova followed by Tukey test post hoc analysis was conducted. Anova II performed on EOG responses revealed genotype and odor as main effects (genotype: *F*_(2,128)_ = 27.18, *p* = 1.46e^−10^, odor: *F*_(1,128)_ = 6.58, *p* = 1.10e^−2^; *p* = *0.05, **0.01, ***0.001). (c, d) AQP4 isoforms do not affect the kinetics of the response (data are presented as mean and ci. *t*_20_: 20% decay time, Anova I and Tukey test post hoc analysis was performed; *WT* = 41 mice in total, *AQP4ex‐KO* = 43, *OAP‐null* = 50). (e) EOG responses to 100 ms exposure to geraniol, eucaliptol, and heptaldehyde, all at 10^−1^ M were recorded from three positions (p1, p2, p3) of turbinate IIa. Peaks are normalized to isoamyl acetate; data are presented as mean ± SE. Linear mixed model performed on normalized EOG responses revealed odorant as main significant effect (odorant: *F*_(2,190.3)_ = 353.19, *p* = 2.2e^−16^) and there were no differences between the three genotypes (*WT* = 6–9 mice, *AQP4ex‐KO* = 6–9, *OAP‐null* = 11–12). EUC: eucaliptol, GER: geraniol, HEP: heptaldehyde, IAA: isoamyl acetate.

A direct consequence of the decrease in OSN density in the OE may be a reduction in the magnitude of the odorant response. A previous report described a decreased EOG response due to altered coupling between water exchange and potassium clearance in the ECS of the OE (Lu et al. 2008). We performed air phase EOG recordings from the OE of WT, AQP4ex‐KO, and OAP‐null mice. When stimulated with isoamyl acetate (IAA) at 10^−1^ M, we observed a response that averaged around −14 mV in WT, which similarly decreased to about half in both AQP4ex‐KO and OAP‐null mice (Figure 7a,b). We stimulated the OE with different odorants, such as geraniol (GER) (Figure 7b), and again observed a decrease in response amplitude in KO animals. Thus, the reduced response amplitude was not odorant dependent. The lack of differences in EOG response kinetics further strengthens the idea that single cell OSN functionality could be unaltered in the AQP4ex‐KO and OAP‐null mice. Indeed, time to peak and decay time (*t*_20_) are similar between the three genotypes (Figure 7c,d). In this scenario, the action of the AQP4 isoforms is probably maintaining the proper OE structural integrity rather than directly the OSN functionality.

**FIGURE 8:**
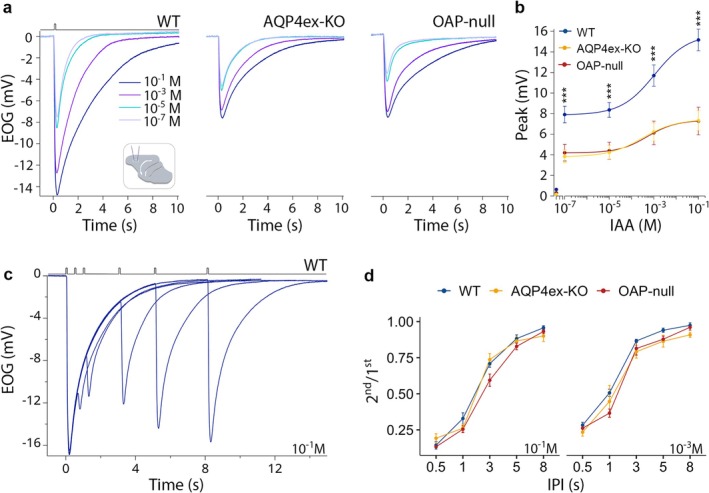
EOG responses from AQP4‐KO mice, recover from adaptation. (a) Odorant‐evoked EOG responses were evoked by 100 ms stimulation with isoamyl acetate vapor of increasing concentrations ranging from 10^−1^ to 10^−7^ M recorded from turbinate IIa of WT, AQP4ex‐KO, and OAP‐null mice. (b) EOG amplitude responses are reduced in KO models. EOG responses to air are also shown; data are presented as mean ± SE. A linear mixed model performed on EOG responses to different concentrations revealed genotype, dose, and genotype‐dose interaction as significant effects (genotype: *F*_(2,28.1)_ = 15.29, *p* = 3.22e^−5^, dose: *F*_(3,77.7)_ = 92.62, *p* = 2.2e^−16^, genotype‐dose: *F*_(6,77.7)_ = 4.42, *p* = 6.80e^−4^; *p* = *0.05, **0.01, ***0.001), nonetheless, there is no difference in genotype‐dose when EOG responses are normalized to the higher dose used; *WT* = 9 mice, *AQP4ex‐KO* = 11, *OAP‐null* = 9. (c) Paired pulse odorant responses evoked by 100 ms stimulation with different interpulse intervals (IPI: 0.5, 1, 3, 5, 8 s) to 10^−1^ M isoamyl acetate (IAA) from WT. (d) Ratio of response to the second stimulus to the first one ± SEM at the indicated odorant concentration plotted versus the IPI; data are presented as mean ± SE. A linear mixed model performed on the 2nd/1st response revealed ipi, dose, and ipi‐dose interaction as main significant effects (ipi: *F*_(4,149.9)_ = 787.94, *p* = 2.2e^−16^, dose: *F*_(1,153.3)_ = 69.49, *p* = 4.09e^−14^, ipi‐dose: *F*_(4,149.9)_ = 7.54, *p* = 1.46e^−5^); *WT* = 7 mice, *AQP4ex‐KO* = 6, *OAP‐null* = 8.

Furthermore, we selected three odorants with different air‐mucus odorant partition coefficients (see methods and Kurtz et al. 2004; Scott et al. 2014; Coppola et al. 2017) and found that we could still observe a significant reduction in the responses of the KO models compared to WT. Normalizing the responses to IAA, heptaldehyde (HEP) consistently elicited higher responses than eucalyptol (EUC) and GER in all models. Importantly, the response profile in KO mice was similar to WT (Figure 7e), suggesting that mucus water content likely remains unchanged in the KO models. Moreover, the dose–response indicated that the amplitude of the response at each tested concentration was smaller in the KO mouse models compared to the WT (Figure 8a,b), although we did not observe a clear shift in odorant sensitivity in either KO mouse models.

A further confirmation that OSN response is not altered comes from the paired‐pulse paradigm experiments to explore adaptation. We applied a pulse of IAA of 100 ms each, with the time between pulses (interpulse intervals, IPI) varying from 0.5 to 8 s (Figure 8c,d). Since the response to the first odor pulse did not decay to baseline when the second pulse was applied, the recorded second response represents the sum of the residual first response and the adapted response to the second pulse. We found no significant differences between the genotypes. Altogether, these results show that the electrophysiological properties of OSNs remain intact, while the overall response amplitude is compromised due to the loss of functional support and proliferative abilities provided by the expression of AQP4 in the different cell types of the OE.

### 2.4 Olfactory‐Driven Behaviors Are Impaired in AQP4ex‐KO and OAP‐Null Mouse Model

**FIGURE 9:**
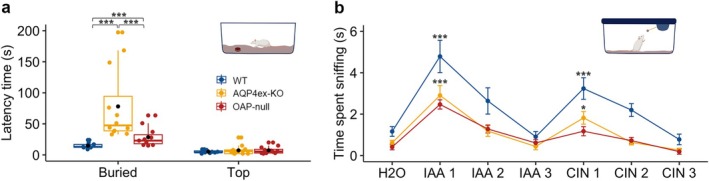
AQP4 isoforms affect olfactory abilities and discrimination. (a) Latency time in finding the cookie buried or placed on top of the bedding. AQP4ex‐KO and OAP‐null mice are slower than WT in finding the cookie. The central point in the box plots represents the mean, central line: Median, upper and lower box boundaries: 25th and 75th percentile, extreme lines: The highest and lowest value. Kruskal–Wallis test followed by Benjamini‐Hochberg (BH) post hoc analysis was performed on buried cookie: *H*_(2)_ = 21.74, *p* = 1.91e^−5^; *p* = *0.05, **0.01, ***0.001; *WT* = 10 mice, *AQP4ex‐KO* = 12, *OAP‐null* = 12. (b) Time spent sniffing the new odorant within 2 min after the odorant was previously presented. IAA and CIN were used at 1:100. A linear mixed model revealed genotype and odorant as main significant effects (genotype: *F*_(2,72.1)_ = 30.71, *p* = 4.354e^−14^, odorant: *F*_(6,182.8)_ = 30.77, *p* = 2.2e^−16^). The statistical differences are to be interpreted as the difference between H_2_O and IAA1, and IAA3 and CIN1 for every genotype. There are no differences between IAA3 and CIN1 in OAP‐null; instead, AQP4ex‐KO is different from WT (*p* = *0.05). Data are presented as mean ± SE; *WT* = 10 mice, *AQP4ex‐KO* = 10, *OAP‐null* = 10. CIN: 1,4 cineole, IAA: isoamyl acetate.

We sought to investigate how the observed changes combine to alter naïve behavior such as tracking odors to find hidden food. We performed the odor‐guided food‐seeking test consisting of a hidden piece of cookie under the cage bedding. The mouse was free to explore the cage, and we measured the latency time to find the cookie. This behavior mainly depends on the olfactory abilities of the mouse. AQP4ex‐KO and OAP‐null mice took longer to find the cookie than WT mice. Three out of 15 AQP4ex‐KO and OAP‐null mice did not locate the cookie within 5 min and were excluded from the study. In contrast, all the tested WT mice found the cookie (Figure 9a). Interestingly, although we did not observe differences in the molecular and electrophysiological properties between the AQP4ex‐KO and OAP‐null models, we found that the former was slower in the cookie test than the latter. We did not find differences between genotypes when the cookie was placed on top of the bedding, allowing the animal to easily reach it without relying on olfaction to find the cookie.

We also analyzed mouse behavior by using the habituation/dishabituation test (Figure 9b). Habituation is the process whereby the mouse's response to a stimulus decreases with repeated exposures, representing nonassociative learning. We presented IAA to the mouse, and this step was repeated three times, and for each iteration, the length of time that the animal spent sniffing the odor source was noted. A reduction in sniffing time over successive trials is interpreted as evidence of the animal recognizing the odor. To assess odor dishabituation, we introduced cineole (CIN) at the fourth trial. The observed increase in investigation time indicates the ability of the mouse to differentiate between the previous (IAA) and new odor (CIN). The three mouse models we tested adapted to the repeated exposures to IAA and CIN. Although the AQP4ex‐KO mice showed a reduced time spent sniffing the new odor (CIN 1) compared to the WT, they increased their investigation time when the new odor was presented. Instead, the OAP‐null mice failed to recognize the new odor, as indicated by the lack of difference in investigation time between the odor they had already sniffed (IAA 3) and the new odor (CIN 1) (Figure 9b). In summary, we could dissect how structural and functional impairments affected olfactory behavior by finding differences between AQP4ex‐KO and OAP‐null. The former was the slowest in finding the cookie, and the latter was unable to discriminate between different odors.

## 3 Discussion

The role of the SUSs in the OE has long been neglected even though they are known to perform various functions, ranging from absorption, detoxification, metabolism, nourishment, phagocytosis, physical support, and secretion (Chen et al. 1992; Getchell and Getchell 1992; Suzuki et al. 1996; Hansen et al. 1998; Hegg et al. 2003). SUSs in the OE abundantly express AQP4 (Figures 1, 2, and 4; Lu et al. 2008). Similarly, AQP4 is widely expressed in sensory systems, such as the inner ear, retina, and vomeronasal organ (Ablimit et al. 2008; Sakai et al. 2014; Maurya et al. 2015; Amann et al. 2016). AQP4 is expressed in the organ of Corti, where the protein seems to play an essential role in inner ear fluid dynamics (Eckhard et al. 2012; Morris et al. 2012; Gleiser et al. 2016). Here, we investigated for the first time the expression of the different AQP4 isoforms and their contribution to the OE physiology.

### 3.1 AQP4ex and AQP4M23 Isoforms Ensure the Structural and Functional Integrity of the Olfactory Epithelium

Since AQP4 can undergo various translational modifications, different isoforms of the protein exist, and depending on the chosen translational start point, AQP4M1 or AQP4M23 can be produced (Neely et al. 1999; Rossi et al. 2010; De Bellis et al. 2017). These two isoforms exhibit a similar water permeability, even though the aggregation to generate OAPs and cellular distribution in the glial cells differ (Crane et al. 2008; Ciappelloni et al. 2019). For example, AQP4M23 and AQP4M1 arrange into OAPs of varying sizes, resulting in increased water permeability (Silberstein et al. 2004; Crane et al. 2009; Rossi et al. 2010). Several studies highlight the connection between K^+^ uptake by astrocytes and water permeability through AQP4. The K^+^‐water coupling hypothesis suggests that K^+^ uptake by astrocytes following neuroexcitation causes osmotic water influx, resulting in astrocyte swelling and ECS shrinkage, which increases K^+^ concentration and enhances its uptake (Amiry‐Moghaddam et al. 2003; Binder et al. 2006; Strohschein et al. 2011; Jin et al. 2012). Thus, astrocytes can maintain an optimal ionic microenvironment in the ECS through AQP4, organized in OAPs, by regulating water balance and allowing neurons to maintain the correct ion concentration for neuroexcitation. AQP4M23 is particularly important for ensuring these mechanisms, as previously noted, because the kinetics of water permeability changes when tested in primary cultures of astrocytes lacking AQP4M23 (Nicchia et al. 2003; Solenov et al. 2004; De Bellis et al. 2021).

As already demonstrated in the brain (De Bellis et al. 2021), here we show that AQP4 staining in the OE is nearly absent when AQP4M23 is missing. This suggests that the aggregation of AQP4 into OAPs is crucial for maintaining normal AQP4 expression levels, and that AQP4M1 alone is not sufficient to maintain the AQP4 channel localization on the basolateral membrane of the SUSs and BCs. Consequently, these cells cannot depend on AQP4 tetramers as an alternative to OAPs. Proper supramolecular organization is crucial either for maintaining the correct extracellular volume microenvironment or for enabling the cell to structurally adapt to the ever‐changing conditions in the OE.

AQP4ex isoform is expressed at the perivascular astrocyte end‐feet (Palazzo et al. 2020), and it is necessary for the perivascular localization of AQP4 isoforms (Palazzo et al. 2019, 2020) and BBB integrity (Mueller et al. 2023). Consequently, AQP4 is no longer localized in the astrocyte end‐feet facing blood vessels in AQP4ex‐KO (Palazzo et al. 2019). In the OE, the lack of the extended isoforms does not affect the expression and localization of the other AQP4 isoforms in the SUSs and BCs, as it occurs in perivascular processes of astrocytes in the brain (Palazzo et al. 2019). Although it has been shown that the lack of AQP4 did not impair OE morphology (Lu et al. 2008), detailed cell counting, as well as isoform‐specific characterization, was not directly attempted. Since EOG measures the summated generator potential from the responding OSNs, the reduced OSN density observed in both transgenic mice would explain the reduced amplitude of EOG responses, without affecting the kinetics of the odorant response. These results suggest the involvement of AQP4ex and OAPs in preserving OE integrity rather than a direct contribution to the OSN transduction machinery. Furthermore, AQP4 most likely does not contribute to setting the composition of the mucus by altering its water content in the OE since odorants with different mucus solubility have similar responses in AQP4ex‐KO and OAP‐null. The basolateral localization of AQP4 in the SUSs further supports this idea.

We found that different cell types (SUSs, proliferating GBCs, and OSNs) have a lower density in the OE of AQP4ex‐KO and OAP‐null mice than WT. Among these, SUSs and GBCs express AQP4 isoforms, while OSNs do not. We can envision a scenario where neurogenesis in the OE is altered. HBCs can drive neurogenesis (Ducray et al. 2002; Conley et al. 2003; Joiner et al. 2015; Gadye et al. 2017) and may involve sensing chemical cues released from the SUSs cells. This is possible because the SUSs' end‐feet surround and intimately contact the BCs (Figure 1). Since AQP4 is highly expressed in the HBCs and SUSs (Figure 4), we hypothesize that AQP4 is necessary to maintain the proper microenvironment. This could directly explain the reduced number of proliferating cells, SUSs, and OSNs, as well as the unchanged number of SOX2^+^‐Ki67^−^ BCs observed in our mouse models. Immunostaining reveals that AQP4 is expressed in the OECs beneath the BL in the OE. Specifically, AQP4M23 is expressed here, since in OAP‐null model, the staining is absent, while AQP4ex‐KO maintains the same staining below the BL. However, AQP4ex staining is minimally detectable beneath the BL (Figure 3). The OECs seem to be involved in roles such as olfactory development, phagocytosis, and responding to neuroinflammation by reducing microglial activation and releasing proinflammatory factors, thereby ameliorating the detrimental conditions of the microenvironment (Lipson et al. 2003; Pastrana et al. 2006; Leung et al. 2008; Rojas‐Mayorquín et al. 2008; Doncel‐Pérez et al. 2009; Zhang et al. 2021). We reasoned that the role played by OECs might be mediated by AQP4M23, favoring OAPs localization in those cells, which may provide structural support and extend OECs' processes that wrap the axons, while the SUSs do this for the cell body and dendrite of the OSNs, aiding in the formation of connections with the OB.

### 3.2 AQP4ex and AQP4M23 Isoforms Impact Olfactory Detection and Discrimination

The odor‐guided food‐seeking test is a naïve behavioral assessment used to evaluate the olfactory ability of mice to locate hidden food sources beneath cage bedding. It is widely utilized, with the time taken by the mouse to find the food depending on a functionally intact olfactory system. The role of the peripheral olfactory system in this behavior is crucial, as altered transduction in the OSNs affects the test (Klein et al. 1996; Pietra et al. 2016). Since the test does not rely on operant conditioning, mice must use their innate sense of smell to identify the odor source for food. AQP4‐KO mice have been shown to take longer to locate food than WT mice (Lu et al. 2008). Here, we expanded on this finding by demonstrating that both AQP4ex‐KO and OAP‐null mice are slower than WT mice, suggesting that both isoforms are essential for maintaining olfactory capabilities, as indicated by the EOG results. We further analyzed the altered behavior, discovering differences between the two transgenic mouse models. AQP4ex‐KO mice exhibited a reduced ability to detect the odor source compared to OAP‐null mice. Nevertheless, the EOG response amplitude is similar in both KO mouse models, indicating that olfactory processing might be affected in the OB or higher olfactory circuits. Thus, different behaviors may suggest that additional changes occur further along in olfactory processing within the brain, potentially explaining the varied behaviors we observed.

The habituation test demonstrated that both AQP4ex‐KO and OAP‐null mouse models could adapt similarly to WT. Interestingly, after behavioral habituation, the OAP‐null mice failed to dishabituate and spent less time than WT and AQP4ex‐KO mice investigating the new odorant. A possible explanation for this behavior is that the altered water dynamics may change the ECS and likely affect basolateral ionic homeostasis. While this alteration may not significantly impact ciliary olfactory transduction, it could impair the firing of action potentials.

The use of two different animal models provided valuable insights into the expression and localization of AQP4 in the OE. In the OAP‐null mouse model, the absence of OAPs leads to a significant reduction of AQP4, effectively creating a condition similar to AQP4‐KO. Although AQP4ex‐KO maintains physiological levels of AQP4 in the OE without showing altered membrane organization, both models demonstrate similar detrimental effects on the OE, such as decreased cell density and impaired olfactory functions.

Overall, we demonstrated that the contribution of the SUSs in maintaining the microenvironment surrounding OSNs and other cell types of the OE could be altered when certain AQP4 isoforms are absent. Furthermore, we aimed to explore how the different AQP4 isoforms affect behavior and their role in olfactory detection and discrimination, discovering that olfactory abilities are compromised. Finally, it is worth noticing that the deletion of AQP4ex (representing only about 6% of total AQP4 in the OE) led to alterations remarkably similar to those observed in the absence of the major isoform AQP4M23. This finding highlights a crucial role for AQP4ex in maintaining an appropriate microenvironment required for proper olfactory function, suggesting that even minor isoforms can exert disproportionately significant effects on sensory physiology. Therefore, our results provide a foundation for further understanding the specific involvement of AQP4 isoforms in the OB and other olfactory circuits.

## 4 Methods

### 4.1 Animals

Mice were maintained under a light/dark cycle (12/12 h) in the Department of Translational Biomedicine and Neuroscience's animal facility at room temperature (22°C ± 2°C) and 75% humidity, with food and water ad libitum. All experiments were performed using procedures approved by the Institutional Committee on Animal Research and Ethics of the University of Bari and the Italian Health Department (Project n°475/2020‐PR) and in accordance with the European directive on animal use for research. Experiments were carried out on mice from 1 to 6 months old and of either sex. All experiments were designed to minimize the number of animals used and their suffering.

C57BL/6J OAP‐null and AQP4ex‐KO mice were generated by Cyagen Biosciences Inc. (Santa Clara, USA) using CRISPR/Cas9 technology as previously described (Palazzo et al. 2019; De Bellis et al. 2021). Specifically, the OAP‐null mouse model was generated by introducing a point mutation (ATG → ATC) in C57BL/6J mice, resulting in a knock‐in mouse with an M23I substitution in the AQP4 locus within the coding sequence of Exon1. Two strains were generated to introduce the isoleucine (ATC) amino acid substitution into the methionine 23 (ATG). This was achieved using two distinct gRNAs (gRNA1 and gRNA2) targeting different regions of the AQP4 sequence. gRNA1: the G → C mutation aligns with the final position of the guide. In order to prevent re‐binding and re‐cutting of the sequence after homology‐directed repair, coinjection with an oligo donor containing an additional silent mutation in the proto spacer‐adjacent motif (PAM) sequence (TCC → TCA) was performed. gRNA2: the G → C mutation occurred in the middle of the guide sequence. gRNA2 was coinjected with an oligo donor without any modification in the PAM sequence.

AQP4ex‐KO mouse model was generated by preventing the translation of the AQP4ex isoforms. Specifically, AQP4 stop codon (UGA) was modified into the UAA codon, and two additional stop codons (UAG, UGA) were introduced. Two sites for Cas9 activity were identified in the target sequence: (gRNA1: ATTGTCTTCCGTATGACTAGAGG and gRNA2: AGTGCTGTCCTCTAGTCATACGG). Nonetheless, gRNA1 was selected since it had the highest quality score (84) and a low probability of off‐target sites (highest score: 0.9). Cas9 mRNA, donor oligo (with the targeting sequence flanked by 120 bp homologous sequences on both sides) and gRNA (generated by in vitro transcription) were coinjected to generate AQP4ex‐KO mice.

In order to genotype AQP4ex‐KO mice, a 0.5 mm tail was collected, and DNA was extracted using the PCR kit (Thermo Scientific Phire Animal Tissue Direct PCR Kit, Thermo Fisher Scientific, USA). The extracted DNA was amplified by PCR using specific primers:

1. Mouse Aqp4‐F: 5′‐GTTGGACCAATCATGGGCGCT‐3′
2. Mouse Aqp4‐R: 5′‐CCAGCTTCCTCACAGAGGTGTCCA‐3′

The 617 bp PCR products were purified using a QIAEX II Gel Extraction Kit (QIAGEN, Germany). The amplified DNA was digested using the Mae III restriction enzyme (Mae III, *Methanococcus aeolicus* PL‐15/H, Roche, Switzerland) to produce three knock‐in fragments (125, 220, 272 bp), two wild‐type fragments (226 and 391 bp), and five heterozygous fragments (125, 220, 226, 272, 391 bp).

### 4.2 Immunohistochemistry

Mice (2–3 months old) were anesthetized with CO_2_ inhalation and decapitated. The dissected nose was fixed in 4% PFA solution overnight at 4°C and washed in PBS Ca^2+^‐Mg^2+^, pH 7.4. Noses were decalcified in EDTA (0.5 M, pH 8) over several days up to a week at 4°C with sporadic replacement of the buffer. The time of decalcification depends on the degree of mineralization of the bone. After washing in PBS Ca^2+^‐Mg^2+^, the buffer solution was replaced with sucrose at 5%, 10%, and 20% (w/v) in PBS Ca^2+^‐Mg^2+^ for 10 min each. Then, tissues were placed in 30% (w/v) sucrose overnight at 4°C. After washing in the buffer solution, tissues were frozen in optimal cutting medium compound Tissue‐Tek OCT (Sakura, The Netherlands). Coronal sections 14–15 μm thick were cut with a cryostat (CM 1900, Leica) at −20°C and stored on super frost + glass slides at −80°C. After rehydration with PBS Ca^2+^‐Mg^2+^, sections were incubated with 0.5% (w/v) SDS in PBS Ca^2+^‐Mg^2+^ for antigen retrieval, followed by blocking solution 5% (v/v) normal goat serum, 4% (w/v) BSA, 5% (w/v) nonfat dry milk, 0.3% (w/v) Triton X‐100 in PBS Ca^2+^‐Mg^2+^ for 30 min. Slices stained with CYT8 and S100B were incubated with 0.5% (w/v) SDS in PBS Ca^2+^‐Mg^2+^, followed by blocking solution 5% (v/v) donkey serum, 2% (w/v) BSA, 0.1% (w/v) Triton X‐100 in PBS Ca^2+^‐Mg^2+^ for 60 min. Then, the slices were incubated overnight at 4°C with rabbit polyclonal anti‐AQP4 (GenScript Biotech, Piscataway, NJ, USA) diluted 1:2000 in the blocking buffer, rabbit polyclonal anti‐AQP4ex generated against the peptide DSTEGRRDSLDLASC within the AQP4 C‐terminus (1:1000; GenScript Biotech), mouse monoclonal anti‐OMP (1:300; Santa Cruz Biotechnology, TX, USA), rat monoclonal anti‐Sox2 (1:300; eBioscience, Invitrogen, San Diego, USA), rabbit polyclonal anti‐Kir 4.1 (1:100, Alomone Labs, Jerusalem, Israel), rabbit polyclonal anti‐Ki67 (1:200; Abcam, Cambridge, UK), rat polyclonal anti‐CYT8–TROMA 1 (1:100; DSHB), rabbit polyclonal S100B (1:300, Proteintech, San Diego, California). Slices were incubated with AlexaFluor 488 goat anti‐rabbit, AlexaFluor 594 donkey anti‐rabbit, and AlexaFluor 594 goat anti‐mouse at 1:1000, and AlexaFluor 488 donkey anti‐rat (1:500) in 0.2% (w/v) Tween 20, PBS Ca^2+^‐Mg^2+^ (all from Life Technologies, Thermo Fisher Scientific, Carlsbad, California, USA) for 45 min at room temperature. After washing in PBS Ca^2+^‐Mg^2+^, sections were mounted with 1:1 Mowiol (Sigma‐Aldrich, Waltham, MA, USA)—DAPI (Life Technologies, Thermo Fisher Scientific, Waltham, MA, USA). Images were acquired with a confocal laser scanning microscope (TCS SP8, Leica) using a 40×/1.30 or 63×/1.40 HC PL Apo CS2 oil objective at 1024 × 1024p, in whole *z* stack, with step size 0.30 μm and zoom factor 1, and analyzed with Fiji software (Schindelin et al. 2012).

### 4.3 Cell Counting

The number of cells with immunoreactive signal for OMP, Sox2, and Ki67 was counted from 12 areas of coronal sections 15 μm thick, spanning widely separated regions in the OE from the anterior part of turbinate II to the ventral region of turbinate IV. SUSs were counted by considering the apical Sox2^+^ cells, which are the nuclei of the SUSs. GBCs were counted as basal Sox2^+^‐Ki67^+^ cells laying on the BL. Basal Sox2^+^‐Ki67^−^ cells were also counted. These latter could be quiescent GBCs and/or HBCs (*n* = 3 mice, 5–6 months old). Cell density was measured in a 117 (h) × 236 (w) μm^2^ area, which included all the OE thickness. Images were acquired with an LED fluorescence microscope (DM2500, Leica) using a 20X/0.55 HC PL FLUOTAR objective at 1280 × 1024p and a CCD camera (DFC7000‐T, LEICA), then analyzed with Fiji software. Pictures in Figure 5a–c were collected in whole *z* stack, with step size 0.30 μm and zoom factor 1 at maximum intensity projection to better visualize the BCs and the nuclei of the SUSs.

### 4.4 SDS‐PAGE and Western Blotting

Mice (5–6 months old of either sex) were anesthetized with CO_2_ inhalation and decapitated. The head was split midsagitally, and the OE was removed. The specimens were stored in liquid nitrogen. Samples were dissolved in BN buffer (5–7× of the sample volume) (1% (w/v) Triton X‐100, 12 mM (w/v) NaCl, 500 mM (w/v) 6‐aminohexanoic acid, 20 mM (v/v) Bis‐Tris, pH 7.0, 2 mM (w/v) EDTA, 10% (w/v) glycerol) and a Protease Inhibitor Cocktail (Roche, Milan, Italy). Lysis was performed on ice, vortexing the samples every 5 min for 30 min, and then the specimens were centrifuged at 15,000 rpm at 4°C for 45 min. The supernatant was collected and stored at −80°C, and the protein concentration was measured with a BCA Protein Assay Kit (Thermo Scientific, Waltham, Massachusetts). The electrophoresis and immunoblotting were performed as previously described (Nicchia et al. 2009). Briefly, proteins were separated on 13% acrylamide/bis‐acrylamide gel for AQP4 (20 μg), AQP4ex (20 μg) and Kir 4.1 (20 μg) and 15% acrylamide/bis‐acrylamide gel for OMP (35 μg) and S100B (45 μg), and transferred to polyvinylidenedifluoride (PVDF) membranes (Millipore, Burlington, Massachusetts, USA) for immunoblot analysis. Membranes were incubated for 1 h in blocking buffer: 5% (w/v) nonfat dry milk, 1% (w/v) Triton X‐100. Membranes stained with S100B were incubated in 5% (w/v) non‐fat dry milk, 1× (v/v) TBST. Hence, membranes were incubated overnight or for 1.5 h at 4°C in blocking buffer with the following primary antibodies: rabbit polyclonal anti‐AQP4 (1:4000, GenScript Biotech), rabbit polyclonal anti‐AQP4ex (1:2000; GenScript Biotech), mouse monoclonal anti‐OMP (1:1000; Santa Cruz Biotechnology), rabbit polyclonal anti‐Kir 4.1 (1:400, Alomone Labs), rabbit polyclonal anti‐S100B (1:400, Proteintech). Then, they were washed in blocking buffer and incubated with the following peroxidase‐conjugated secondary antibodies at room temperature for 45 min: goat anti‐rabbit IgG HRP (1:3000; Bio‐Rad, California, USA) and goat antimouse IgG HRP (1:3000; Bio‐Rad). Hence, membranes were washed in Triton X‐100 or TBST. Proteins were detected using an enhanced chemiluminescent detection system (Clarity Western ECL Substrate, Bio‐Rad) and visualized with Chemidoc Touch imaging system (Bio‐Rad). Densitometric analysis was performed using Image Lab software (Bio‐Rad). For each lane, the intensity of the analyzed band was first normalized to the total protein loaded, visualized by Ponceau staining. Subsequently, values from all samples were expressed relative to the mean of the WT group and reported as apercentage.

### 4.5 Electro‐Olfactogram Recording

The experimental procedure is similar to that previously described by Zhao et al. (1998), Cygnar et al. (2010), and Guarneri et al. (2023). Mice (5–6 months old) were anesthetized with CO_2_ inhalation and decapitated. The head was split sagittally along the midline and immediately placed on a dissecting microscope (Stemi DV4, Zeiss) on a vibration‐isolating table (Supertech Instruments) and shielded from electrical fields by a Faraday cage. The turbinates were exposed by removing the septum, and the specimen was continuously perfused with bubbled distilled water at 37°C to guarantee the hydration of the epithelium. The recording electrode was made from borosilicate glass (World Precision Instruments) pulled with a P‐1000 puller (Sutter Instrument) and then placed on the surface of one of the turbinates. The pipette was filled with Ringer's solution (mM): 140 NaCl, 5 KCl, 2 CaCl_2_, 1 MgCl_2_, 10 Hepes, 0.01 EDTA, pH 7.4 with NaOH, and the tip of the pipette was filled with 0.5% (w/v) agar in Ringer. The stimulus pulse was controlled by a Picospritzer‐controlled valve (Picospritzer II, Parker Hannifin). A 100 ms pulse of vapor phase odorant was injected at 10 psi into a continuous stream of humidified air. A flow meter (Masterflex) set to 3 L/min was used to control the flow of humidified air arriving at the sample. The device connected the Picospritzer to the delivery tube. The odorant vapor was generated by placing an odorant solution in a 5 mL glass test tube sealed with a rubber stopper. The system used two 20‐gauge needles as input and output ports of the vapor above the solution. The reference electrode was electrically connected to the tissue by positioning it into the mouse brain. The odorants used: IAA, GER, EUC, and HEP, were prepared every day from 5 M stock in dimethyl sulfoxide (DMSO) (Honeywell, Riedel de Haën, Seelze, Germany), then diluted in water to reach a final concentration from 10^−7^ to 10^−1^ M. The log (air/mucus partition coefficients) were obtained from (Kurtz et al. 2004; Scott et al. 2014; Coppola et al. 2017) and were −2.4038 for IAA, −2.766 for heptaldehyde, −3.4368 for EUC, and −5 for GER.

EOG responses were recorded at room temperature. Data were collected with an EPC 10 USB (HEKA, Elektronik) amplifier and analyzed using PatchMaster Next (version 1.4.1). The signals were recorded at a sampling rate of 10 kHz and low‐pass filtered at 2.9 kHz. Kinetics of EOG responses were evaluated, measuring time to peak, decay time, rise time, latency time, and FWHM (full width at half maximum) (data not shown). Time to peak was determined as the time from the start of the stimulation to the peak of the response; decay time (*t*_20_) as the time the response takes to decrease to 20% of the peak; latency as the time between the start of odorant stimulation to 1% of the peak value; the rise time as the time between the start of the response and the peak. All chemicals were purchased from Sigma‐Aldrich.

### 4.6 Single‐Cell RNA Sequencing Analysis

Single‐cell RNA sequencing (scRNA‐seq) dataset was downloaded from NCBI GEO: GSE151346 deposited by Brann et al. (2020). Specifically, Drop‐seq was run on dissociated whole olfactory mucosa from 5 male C57BL/6J mice 2–3 months old. “MOE_all_counts” and “MOE_metadata” files, with the latter containing cell types and UMAP coordinates, were used to represent %AQP4^+^ cells and %AQP4 UMIs in every cell type of the OE. AQP4 expression clustered by cell type was visualized using the provided UMAP coordinates.

### 4.7 Odor‐Guided Food‐Seeking Test

A small piece of food was buried below the surface of the cage bedding so that it was a purely olfactory cue, and the time taken by the mouse to find the food was recorded. All mice of either sex (5–6 months old) were maintained under a light/dark cycle (12/12 h) and tested during the light phase. Mice were food‐deprived overnight with ad libitum access to water. Before the test, each mouse was familiarized with the experimental setting by placement in the enclosure for 5 min and then placed in a novel cage (l × h × w; 30 cm × 12 cm × 15 cm) where a ∼3 g piece of cookie (Oreo) had been buried under ∼2 cm of fresh bedding. The latency of the animal to retrieve the cookie was recorded with a stopwatch. Retrieving the cookie was defined as digging it up with forepaws, picking it up, and placing it in the mouth. We set a 5 min limit within which the animals had to find the cookie. After that, the time was stopped, and the cookie was exposed so the mouse could access and eat it. The position of the buried cookie was randomly changed in each experiment. The next day, the cookie was placed on the bedding. Since just some KO mice could not find the cookie within 5 min, we excluded them.

### 4.8 Habituation/Dishabituation Test

A progressive decrease in olfactory investigation time toward a repeated presentation of the same odor stimulus defines habituation. Dishabituation allows the reinstatement of odor investigation when a novel odor is presented (Woodley and Baum 2003; Wrenn et al. 2004). Therefore, this test assesses if the animal is able to distinguish different odors. The experimental procedure is similar to that previously described by Yang and Crawley (2009). Briefly, before starting the test, each mouse was acclimated for 30 min in a dedicated room and placed in a novel cage (l × h × w; 30 cm × 12 cm × 15 cm) where a clean and dry applicator was inserted through the water bottle hole of the cage lid. This procedure is crucial to reduce novelty‐induced exploratory activity during the olfactory test. Then, each mouse was exposed to sequential presentations of different odors in the following order: water, IAA and GER diluted 1:100. Each odor was presented in three consecutive trials for 2 min, and the time spent sniffing the applicator with the odorant was recorded with a stopwatch. The intertrial interval was 1 min. The cotton tip part of the applicator did not touch any part of the cage lid while changing the odor, avoiding cross‐trial contamination.

### 4.9 Statistical Analysis

Statistical analysis was conducted using R (R Studio 2024). Sample distribution was assessed for normality with the Shapiro–Wilk test using the *shapiro.test()* function, and a parametric or nonparametric test was conducted accordingly. Anova (*anova_test()*) from “*rstatix*” package (Kassambara 2023), followed by the Tukey test post hoc analysis, was used as the parametric test. Kruskal‐Wallis (*kruskal.test()*), followed by the Benjamini‐Hochberg (BH) post hoc analysis (*pairwise.wilcox.test()*) was used as a nonparametric test. All the aforementioned functions were used by running “*stats*” package (R Core Team 2021). The “*emmeans*” package (Lenth 2023) was used for performing pairwise comparisons of estimated marginal means (EMMs) by using the *pairs()* function. EMMs compare the levels of a factor by removing the effects of other factors and were used to interpret the main effect and interactions. Mixed model Anova was used for multilevel analyses. In particular, in the R environment, dependent variables were investigated with linear mixed models (LMMs). LMMs were computed using the *lmer()* function (“*lme4*” package, Bates et al. 2015). Finally, in order to get *F* statistics and *p*‐value for the fixed effects of the models, we ran Anova using “*lmerTest*” package (Kuznetsova et al. 2017). Post hoc comparisons were performed as stated in the figure legends. “*ggplot2*” package (Wickham 2016) was used to create the graphs.

## Author Contributions

D.L. performed electrophysiological and behavioral experiments. D.L. and P.A. performed Western Blotting. D.L. and M.G.F. performed immunofluorescence experiments. D.L., M.G.F., and B.B. performed image acquisition. D.L. and M.D. analyzed the experiments. P.A., O.V., and C.P. collected the initial data. M.D., D.L., A.F., and G.P.N. conceptualized and wrote the manuscript. All the authors approved the final version of the manuscript.

## Ethics Statement

All experiments were performed using procedures approved by the Institutional Committee on Animal Research and Ethics of the University of Bari and the Italian Health Department (Project n°475/2020‐PR) and in accordance with the European directive on animal use for research.

## Conflicts of Interest

The authors declare no conflicts of interest.

## Data Availability

The data that support the findings of this study are available from the first and corresponding authors upon reasonable request.

## References

[glia70024-bib-0001] Ablimit, A. , T. Aoki , T. Matsuzaki , et al. 2008. “Immunolocalization of Water Channel Aquaporins in the Vomeronasal Organ of the Rat: Expression of AQP4 in Neuronal Sensory Cells.” Chemical Senses 33, no. 5: 481–488. 10.1093/chemse/bjn015. 18407959

[glia70024-bib-0002] Ablimit, A. , T. Matsuzaki , Y. Tajika , T. Aoki , H. Hagiwara , and K. Takata . 2006. “Immunolocalization of Water Channel Aquaporins in the Nasal Olfactory Mucosa.” Archives of Histology and Cytology 69, no. 1: 1–12. 10.1679/aohc.69.1. 16609265

[glia70024-bib-0003] Acevedo, C. , K. Blanchard , J. Bacigalupo , and C. Vergara . 2019. “Possible ATP Trafficking by ATP‐Shuttles in the Olfactory Cilia and Glucose Transfer Across the Olfactory Mucosa.” FEBS Letters 593, no. 6: 601–610. 10.1002/1873-3468.13346. 30801684

[glia70024-bib-0004] Afhami Mina, M. , K. Hashemi , J. Afshari , et al. 2018. “Acute Transplantation of Human Olfactory Mucosa‐Derived Olfactory Ensheathing Cells Fails to Improve Locomotor Recovery in Rats.” Acta Medica Iranica 56, no. 7.

[glia70024-bib-0005] Agostinelli, E. , K. Y. Gonzalez‐Velandia , A. Hernandez‐Clavijo , et al. 2021. “A Role for STOML3 in Olfactory Sensory Transduction.” Eneuro 8, no. 2: ENEURO.0565‐20.2021. 10.1523/ENEURO.0565-20.2021. PMC7986538 33637538

[glia70024-bib-0006] Allison, A. C. 1953. “The Morphology of the Olfactory System in the Vertebrates.” Biological Reviews 28, no. 2: 195–244. 10.1111/j.1469-185X.1953.tb01376.x.

[glia70024-bib-0007] Amann, B. , K. J. H. Kleinwort , S. Hirmer , et al. 2016. “Expression and Distribution Pattern of Aquaporin 4, 5 and 11 in Retinas of 15 Different Species.” International Journal of Molecular Sciences 17, no. 7: 1145. 10.3390/ijms17071145. 27438827 PMC4964518

[glia70024-bib-0008] Amiry‐Moghaddam, M. , A. Williamson , M. Palomba , et al. 2003. “Delayed K+ Clearance Associated With Aquaporin‐4 Mislocalization: Phenotypic Defects in Brains of α‐Syntrophin‐Null Mice.” Proceedings of the National Academy of Sciences 100, no. 23: 13615–13620. 10.1073/pnas.2336064100. PMC263862 14597704

[glia70024-bib-0009] Arguello, J. R. , L. Abuin , J. Armida , K. Mika , P. C. Chai , and R. Benton . 2021. “Targeted Molecular Profiling of Rare Olfactory Sensory Neurons Identifies Fate, Wiring, and Functional Determinants.” eLife 10: e63036. 10.7554/eLife.63036. 33666172 PMC7993999

[glia70024-bib-0010] Bates, D. , M. Maechler , B. Bolker , and S. Walker . 2015. “Fitting Linear Mixed‐Effects Models Using lme4.” Journal of Statistical Software 67, no. 1: 1–48. 10.18637/jss.v067.i01.

[glia70024-bib-0011] Binder, D. K. , X. Yao , Z. Zador , T. J. Sick , A. S. Verkman , and G. T. Manley . 2006. “Increased Seizure Duration and Slowed Potassium Kinetics in Mice Lacking Aquaporin‐4 Water Channels.” Glia 53, no. 6: 631–636. 10.1002/glia.20318. 16470808

[glia70024-bib-0012] Brann, D. H. , T. Tsukahara , C. Weinreb , et al. 2020. “Non‐Neuronal Expression of SARS‐CoV‐2 Entry Genes in the Olfactory System Suggests Mechanisms Underlying COVID‐19‐Associated Anosmia.” Science Advances 6, no. 31: eabc5801. 10.1126/sciadv.abc5801. 32937591 PMC10715684

[glia70024-bib-0013] Buck, L. , and R. Axel . 1991. “A Novel Multigene Family May Encode Odorant Receptors: A Molecular Basis for Odor Recognition.” Cell 65, no. 1: 175–187. 10.1016/0092-8674(91)90418-x. 1840504

[glia70024-bib-0014] Chen, Y. , M. L. Getchell , X. Ding , and T. V. Getchell . 1992. “Immunolocalization of Two Cytochrome P450 Isozymes in Rat Nasal Chemosensory Tissue.” Neuroreport 3, no. 9. 10.1097/00001756-199209000-00007 1421130

[glia70024-bib-0015] Ciappelloni, S. , D. Bouchet , N. Dubourdieu , et al. 2019. “Aquaporin‐4 Surface Trafficking Regulates Astrocytic Process Motility and Synaptic Activity in Health and Autoimmune Disease.” Cell Reports 27, no. 13: 3860–3872. 10.1016/j.celrep.2019.05.097. 31242419

[glia70024-bib-0016] Conley, D. B. , A. M. Robinson , M. J. Shinners , and R. C. Kern . 2003. “Age‐Related Olfactory Dysfunction: Cellular and Molecular Characterization in the Rat.” American Journal of Rhinology 17, no. 3: 169–175. 10.1177/194589240301700311. 12862407

[glia70024-bib-0017] Coppola, D. M. , B. E. Ritchie , and B. A. Craven . 2017. “Tests of the Sorption and Olfactory “Fovea” Hypotheses in the Mouse.” Journal of Neurophysiology 118, no. 5: 2770–2788. 10.1152/jn.00455.2017. 28877965 PMC5675904

[glia70024-bib-0018] Crane, J. M. , J. L. Bennett , and A. S. Verkman . 2009. “Live Cell Analysis of Aquaporin‐4 M1/M23 Interactions and Regulated Orthogonal Array Assembly in Glial Cells.” Journal of Biological Chemistry 284, no. 51: 35850–35860. 10.1074/jbc.M109.071670. 19843522 PMC2791014

[glia70024-bib-0019] Crane, J. M. , A. N. Van Hoek , W. R. Skach , and A. S. Verkman . 2008. “Aquaporin‐4 Dynamics in Orthogonal Arrays in Live Cells Visualized by Quantum Dot Single Particle Tracking.” Molecular Biology of the Cell 19, no. 8: 3369–3378. 10.1091/mbc.e08-03-0322. 18495865 PMC2488293

[glia70024-bib-0020] Cygnar, K. D. , A. B. Stephan , and H. Zhao . 2010. “Analyzing Responses of Mouse Olfactory Sensory Neurons Using the Air‐Phase Electroolfactogram Recording.” Journal of Visualized Experiments 37, no. 37: e1850. 10.3791/1850. PMC3125698 20197755

[glia70024-bib-0021] De Bellis, M. , A. Cibelli , M. G. Mola , et al. 2021. “Orthogonal Arrays of Particle Assembly Are Essential for Normal Aquaporin‐4 Expression Level in the Brain.” Glia 69, no. 2: 473–488. 10.1002/glia.23909. 32946135

[glia70024-bib-0022] De Bellis, M. , F. Pisani , M. G. Mola , et al. 2017. “Translational Readthrough Generates New Astrocyte AQP4 Isoforms That Modulate Supramolecular Clustering, Glial Endfeet Localization, and Water Transport.” Glia 65, no. 5: 790–803. 10.1002/glia.23126. 28206694

[glia70024-bib-0023] Doncel‐Pérez, E. , S. Caballero‐Chacón , and M. Nieto‐Sampedro . 2009. “Neurosphere Cell Differentiation to Aldynoglia Promoted by Olfactory Ensheathing Cell Conditioned Medium.” Glia 57, no. 13: 1393–1409. 10.1002/glia.20858. 19235256

[glia70024-bib-0024] Dooley, R. , A. Mashukova , B. Toetter , H. Hatt , and E. M. Neuhaus . 2011. “Purinergic Receptor Antagonists Inhibit Odorant‐Mediated CREB Phosphorylation in Sustentacular Cells of Mouse Olfactory Epithelium.” BMC Neuroscience 12, no. 1: 86. 10.1186/1471-2202-12-86. 21859486 PMC3176191

[glia70024-bib-0025] Duan, S. , C. M. Anderson , E. C. Keung , Y. Chen , Y. Chen , and R. A. Swanson . 2003. “P2X7 Receptor‐Mediated Release of Excitatory Amino Acids From Astrocytes.” Journal of Neuroscience: The Official Journal of the Society for Neuroscience 23, no. 4: 1320–1328. 10.1523/JNEUROSCI.23-04-01320.2003. 12598620 PMC6742264

[glia70024-bib-0026] Ducray, A. , J.‐R. Bondier , G. Michel , K. Bon , A. Propper , and A. Kastner . 2002. “Recovery Following Peripheral Destruction of Olfactory Neurons in Young and Adult Mice.” European Journal of Neuroscience 15, no. 12: 1907–1917. 10.1046/j.1460-9568.2002.02044.x. 12099897

[glia70024-bib-0027] Eckhard, A. , C. Gleiser , H. Rask‐Andersen , et al. 2012. “Co‐Localisation of Kir4.1 and AQP4 in Rat and Human Cochleae Reveals a Gap in Water Channel Expression at the Transduction Sites of Endocochlear K+ Recycling Routes.” Cell and Tissue Research 350, no. 1: 27–43. 10.1007/s00441-012-1456-y. 22802001

[glia70024-bib-0028] Frigeri, A. , M. A. Gropper , M. Fuminori Umenishi , D. B. Kawashima , and A. S. Verkman . 1995. “Localization of MIWC and GLIP Water Channel Homologs in Neuromuscular, Epithelial and Glandular Tissues.” Journal of Cell Science 108, no. 9: 2993–3002. 10.1242/jcs.108.9.2993. 8537439

[glia70024-bib-0029] Frontera, J. L. , A. S. Cervino , L. D. Jungblut , and D. A. Paz . 2015. “Brain‐Derived Neurotrophic Factor (BDNF) Expression in Normal and Regenerating Olfactory Epithelium of *Xenopus laevis* .” Annals of Anatomy ‐ Anatomischer Anzeiger 198: 41–48. 10.1016/j.aanat.2014.10.010. 25488259

[glia70024-bib-0030] Fukuyama, Y. , K. Okada , M. Yamaguchi , H. Kiyono , K. Mori , and Y. Yuki . 2015. “Nasal Administration of Cholera Toxin as a Mucosal Adjuvant Damages the Olfactory System in Mice.” PLoS One 10: e0139368. 10.1371/journal.pone.0139368. 26422280 PMC4589288

[glia70024-bib-0031] Furman, C. S. , D. A. Gorelick‐Feldman , K. G. V. Davidson , et al. 2003. “Aquaporin‐4 Square Array Assembly: Opposing Actions of M1 and M23 Isoforms.” Proceedings of the National Academy of Sciences 100, no. 23: 13609–13614. 10.1073/pnas.2235843100. PMC263861 14597700

[glia70024-bib-0032] Gadye, L. , D. Das , M. A. Sanchez , et al. 2017. “Injury Activates Transient Olfactory Stem Cell States With Diverse Lineage Capacities.” Cell Stem Cell 21, no. 6: 775–790.e9. 10.1016/j.stem.2017.10.014. 29174333 PMC5728414

[glia70024-bib-0033] Getchell, M. L. , and T. V. Getchell . 1992. “Fine Structural Aspects of Secretion and Extrinsic Innervation in the Olfactory Mucosa.” Microscopy Research and Technique 23, no. 2: 111–127. 10.1002/jemt.1070230203. 1421551

[glia70024-bib-0034] Gleiser, C. , A. Wagner , P. Fallier‐Becker , H. Wolburg , B. Hirt , and A. F. Mack . 2016. “Aquaporin‐4 in Astroglial Cells in the CNS and Supporting Cells of Sensory Organs – A Comparative Perspective.” International Journal of Molecular Sciences 17, no. 9: 1411. 10.3390/ijms17091411. 27571065 PMC5037691

[glia70024-bib-0035] Guarneri, G. , S. Pifferi , M. Dibattista , J. Reisert , and A. Menini . 2023. “Paradoxical Electro‐Olfactogram Responses in TMEM16B Knock‐Out Mice.” Chemical Senses 48, no. 2023: bjad003. 10.1093/chemse/bjad003. 36744918 PMC9951260

[glia70024-bib-0036] Guo, Z. , A. Packard , R. C. Krolewski , M. T. Harris , G. L. Manglapus , and J. E. Schwob . 2010. “Expression of Pax6 and Sox2 in Adult Olfactory Epithelium.” Journal of Comparative Neurology 518, no. 21: 4395–4418. 10.1002/cne.22463. 20852734 PMC2940252

[glia70024-bib-0037] Hansel, D. E. , B. A. Eipper , and G. V. Ronnett . 2001. “Neuropeptide Y Functions as a Neuroproliferative Factor.” Nature 410, no. 6831: 940–944. 10.1038/35073601. 11309620

[glia70024-bib-0038] Hansen, A. , J. O. Reiss , C. L. Gentry , and G. D. Burd . 1998. “Ultrastructure of the Olfactory Organ in the Clawed Frog, *Xenopus laevis*, During Larval Development and Metamorphosis.” Journal of Comparative Neurology 398, no. 2: 273–288. 10.1002/(SICI)1096-9861(19980824)398:2<273::AID-CNE8>3.0.CO;2-Y. 9700571

[glia70024-bib-0039] Hasegawa, H. , T. Ma , W. Skach , M. A. Matthay , and A. S. Verkman . 1994. “Molecular Cloning of a Mercurial‐Insensitive Water Channel Expressed in Selected Water‐Transporting Tissues.” Journal of Biological Chemistry 269, no. 8: 5497–5500. 10.1016/S0021-9258(17)37486-0. 7509789

[glia70024-bib-0040] Hayoz, S. , C. Jia , and C. C. Hegg . 2012. “Mechanisms of Constitutive and ATP‐Evoked ATP Release in Neonatal Mouse Olfactory Epithelium.” BMC Neuroscience 13, no. 1: 53. 10.1186/1471-2202-13-53. 22640172 PMC3444318

[glia70024-bib-0041] Hegg, C. C. , D. Greenwood , W. Huang , P. Han , and M. T. Lucero . 2003. “Activation of Purinergic Receptor Subtypes Modulates Odor Sensitivity.” Journal of Neuroscience 23, no. 23: 8291. 10.1523/JNEUROSCI.23-23-08291.2003. 12967991 PMC2976511

[glia70024-bib-0042] Hegg, C. C. , M. Irwin , and M. T. Lucero . 2009. “Calcium Store‐Mediated Signaling in Sustentacular Cells of the Mouse Olfactory Epithelium.” Glia 57, no. 6: 634–644. 10.1002/glia.20792. 18942758 PMC2657191

[glia70024-bib-0043] Henriques, T. , E. Agostinelli , A. Hernandez‐Clavijo , et al. 2019. “TMEM16A Calcium‐Activated Chloride Currents in Supporting Cells of the Mouse Olfactory Epithelium.” Journal of General Physiology 151, no. 7: 954–966. 10.1085/jgp.201812310. 31048412 PMC6605691

[glia70024-bib-0044] Hernandez‐Clavijo, A. , N. Sarno , K. Y. Gonzalez‐Velandia , et al. 2021. “TMEM16A and TMEM16B Modulate Pheromone‐Evoked Action Potential Firing in Mouse Vomeronasal Sensory Neurons.” Eneuro 8, no. 5: ENEURO.0179‐21.2021. 10.1523/ENEURO.0179-21.2021. PMC8445037 34433575

[glia70024-bib-0045] Hirt, B. , C. Gleiser , A. Eckhard , et al. 2011. “All Functional Aquaporin‐4 Isoforms Are Expressed in the Rat Cochlea and Contribute to the Formation of Orthogonal Arrays of Particles.” Neuroscience 189: 79–92. 10.1016/j.neuroscience.2011.05.037. 21621589

[glia70024-bib-0046] Jang, W. , X. Chen , D. Flis , M. Harris , and J. E. Schwob . 2014. “Label‐Retaining, Quiescent Globose Basal Cells Are Found in the Olfactory Epithelium.” Journal of Comparative Neurology 522, no. 4: 731–749. 10.1002/cne.23470. 24122672 PMC4240005

[glia70024-bib-0047] Jin, B.‐J. , A. Rossi , and A. S. Verkman . 2011. “Model of Aquaporin‐4 Supramolecular Assembly in Orthogonal Arrays Based on Heterotetrameric Association of M1‐M23 Isoforms.” Biophysical Journal 100, no. 12: 2936–2945. 10.1016/j.bpj.2011.05.012. 21689527 PMC3123972

[glia70024-bib-0048] Jin, B.‐J. , H. Zhang , D. K. Binder , and A. S. Verkman . 2012. “Aquaporin‐4–Dependent K+ and Water Transport Modeled in Brain Extracellular Space Following Neuroexcitation.” Journal of General Physiology 141, no. 1: 119–132. 10.1085/jgp.201210883. PMC3536523 23277478

[glia70024-bib-0049] Joiner, A. M. , W. W. Green , J. C. McIntyre , B. L. Allen , J. E. Schwob , and J. R. Martens . 2015. “Primary Cilia on Horizontal Basal Cells Regulate Regeneration of the Olfactory Epithelium.” Journal of Neuroscience 35, no. 40: 13761. 10.1523/JNEUROSCI.1708-15.2015. 26446227 PMC4595624

[glia70024-bib-0050] Jones, D. T. , and R. R. Reed . 1989. “Golf: An Olfactory Neuron Specific‐G Protein Involved in Odorant Signal Transduction.” Science 244, no. 4906: 790–795. 10.1126/science.2499043. 2499043

[glia70024-bib-0051] Jourdain, P. , L. H. Bergersen , K. Bhaukaurally , et al. 2007. “Glutamate Exocytosis From Astrocytes Controls Synaptic Strength.” Nature Neuroscience 10, no. 3: 331–339. 10.1038/nn1849. 17310248

[glia70024-bib-0052] Jung, J. S. , R. V. Bhat , G. M. Preston , W. B. Guggino , J. M. Baraban , and P. Agre . 1994. “Molecular Characterization of an Aquaporin cDNA From Brain: Candidate Osmoreceptor and Regulator of Water Balance.” Proceedings of the National Academy of Sciences of the United States of America 91, no. 26: 13052–13056. 10.1073/pnas.91.26.13052. 7528931 PMC45579

[glia70024-bib-0053] Kassambara, A. 2023. “rstatix: Pipe‐Friendly Framework for Basic Statistical Tests.” R Package Version 0.7.2. https://CRAN.R‐project.org/package=rstatix.

[glia70024-bib-0054] Kitaura, H. , M. Tsujita , V. J. Huber , et al. 2009. “Activity‐Dependent Glial Swelling Is Impaired in Aquaporin‐4 Knockout Mice.” Neuroscience Research 64, no. 2: 208–212. 10.1016/j.neures.2009.03.002. 19428702

[glia70024-bib-0055] Klein, S. L. , L. J. Kriegsfeld , J. E. Hairston , V. Rau , R. J. Nelson , and P. J. Yarowsky . 1996. “Characterization of Sensorimotor Performance, Reproductive and Aggressive Behaviors in Segmental Trisomic 16 (Ts65Dn) Mice.” Physiology & Behavior 60, no. 4: 1159–1164. 10.1016/0031-9384(96)00218-1. 8884947

[glia70024-bib-0056] Kurtz, D. B. , K. Zhao , D. E. Hornung , and P. Scherer . 2004. “Experimental and Numerical Determination of Odorant Solubility in Nasal and Olfactory Mucosa.” Chemical Senses 29, no. 9: 763–773. 10.1093/chemse/bjh079. 15574812

[glia70024-bib-0057] Kuznetsova, A. , P. B. Brockhoff , and R. H. B. Christensen . 2017. “lmerTest Package: Tests in Linear Mixed Effects Models.” Journal of Statistical Software 82, no. 1: 1–26. 10.18637/jss.v082.i13.

[glia70024-bib-0058] Landis, D. M. D. , and T. S. Reese . 1974. “Arrays of Particles in Freeze‐Fractured Astrocytic Membranes.” Journal of Cell Biology 60, no. 1: 316–320. 10.1083/jcb.60.1.316. 4809245 PMC2109126

[glia70024-bib-0059] Lenth, R. V. 2023. “emmeans: Estimated Marginal Means, aka Least‐Squares Means.” R Package Version 1.8.7. https://CRAN.R‐project.org/package=emmeans.

[glia70024-bib-0060] Leung, J. Y. K. , J. A. Chapman , J. A. Harris , et al. 2008. “Olfactory Ensheathing Cells Are Attracted to, and Can Endocytose, Bacteria.” Cellular and Molecular Life Sciences 65, no. 17: 2732–2739. 10.1007/s00018-008-8184-1. 18604629 PMC11131851

[glia70024-bib-0061] Li, Y.‐K. , F. Wang , W. Wang , et al. 2012. “Aquaporin‐4 Deficiency Impairs Synaptic Plasticity and Associative Fear Memory in the Lateral Amygdala: Involvement of Downregulation of Glutamate Transporter‐1 Expression.” Neuropsychopharmacology 37, no. 8: 1867–1878. 10.1038/npp.2012.34. 22473056 PMC3376319

[glia70024-bib-0062] Liang, F. 2018. “Olfactory Receptor Neuronal Dendrites Become Mostly Intra‐Sustentacularly Enwrapped Upon Maturity.” Journal of Anatomy 232, no. 4: 674–685. 10.1111/joa.12777. 29313978 PMC5835782

[glia70024-bib-0063] Lipson, A. C. , J. Widenfalk , E. Lindqvist , T. Ebendal , and L. Olson . 2003. “Neurotrophic Properties of Olfactory Ensheathing Glia.” Experimental Neurology 180, no. 2: 167–171. 10.1016/S0014-4886(02)00058-4. 12684030

[glia70024-bib-0064] Loughran, G. , M.‐Y. Chou , I. P. Ivanov , et al. 2014. “Evidence of Efficient Stop Codon Readthrough in Four Mammalian Genes.” Nucleic Acids Research 42, no. 14: 8928–8938. 10.1093/nar/gku608. 25013167 PMC4132726

[glia70024-bib-0065] Lu, D. C. , H. Zhang , Z. Zador , and A. S. Verkman . 2008. “Impaired Olfaction in Mice Lacking Aquaporin‐4 Water Channels.” FASEB Journal 22, no. 9: 3216–3223. 10.1096/fj.07-104836. 18511552 PMC2518258

[glia70024-bib-0066] Lu, M. , M. D. Lee , B. L. Smith , et al. 1996. “The Human AQP4 Gene: Definition of the Locus Encoding Two Water Channel Polypeptides in Brain.” Proceedings of the National Academy of Sciences of the United States of America 93, no. 20: 10908–10912. 10.1073/pnas.93.20.10908. 8855281 PMC38256

[glia70024-bib-0067] Maurya, D. K. , T. Henriques , M. Marini , et al. 2015. “Development of the Olfactory Epithelium and Nasal Glands in TMEM16A−/− and TMEM16A+/+ Mice.” PLoS One 10, no. 6: e0129171. 10.1371/journal.pone.0129171. 26067252 PMC4465891

[glia70024-bib-0068] Morris, L. M. , J. M. DeGagne , J. B. Kempton , F. Hausman , and D. R. Trune . 2012. “Mouse Middle Ear Ion Homeostasis Channels and Intercellular Junctions.” PLoS One 7, no. 6: e39004. 10.1371/journal.pone.0039004. 22720014 PMC3376096

[glia70024-bib-0069] Morrison, E. E. , and R. M. Costanzo . 1992. “Morphology of Olfactory Epithelium in Humans and Other Vertebrates.” Microscopy Research and Technique 23, no. 1: 49–61. 10.1002/jemt.1070230105. 1392071

[glia70024-bib-0070] Mueller, S. M. , K. M. F. White , S. B. Fass , et al. 2023. “Evaluation of Gliovascular Functions of AQP4 Readthrough Isoforms.” Frontiers in Cellular Neuroscience 17. 10.3389/fncel.2023.1272391. PMC10701521 38077948

[glia70024-bib-0071] Nedergaard, M. , B. Ransom , and S. A. Goldman . 2003. “New Roles for Astrocytes: Redefining the Functional Architecture of the Brain.” Trends in Neurosciences 26, no. 10: 523–530. 10.1016/j.tins.2003.08.008. 14522144

[glia70024-bib-0072] Neely, J. D. , B. M. Christensen , S. Nielsen , and P. Agre . 1999. “Heterotetrameric Composition of Aquaporin‐4 Water Channels.” Biochemistry 38, no. 34: 11156–11163. 10.1021/bi990941s. 10460172

[glia70024-bib-0073] Nicchia, G. P. , B. Nico , L. M. A. Camassa , et al. 2004. “The Role of Aquaporin‐4 in the Blood–Brain Barrier Development and Integrity: Studies in Animal and Cell Culture Models.” Neuroscience 129, no. 4: 935–944. 10.1016/j.neuroscience.2004.07.055. 15561409

[glia70024-bib-0074] Nicchia, G. P. , A. Rossi , M. G. Mola , et al. 2010. “Higher Order Structure of Aquaporin‐4.” Aquaporins in the Brain and Spinal Cord 168, no. 4: 903–914. 10.1016/j.neuroscience.2010.02.008. 20153404

[glia70024-bib-0075] Nicchia, G. P. , A. Frigeri , G. M. Liuzzi , and M. Svelto . 2003. “Inhibition of AQP4 Expression in Astrocytes by RNAi Determines Alterations in Cell Morphology, Growth, and Water Transport and Induces Changes in Ischemia Related Genes.” FASEB Journal 17, no. 11: 1–21. 10.1096/fj.02-1183fje. 12824287

[glia70024-bib-0076] Nicchia, G. P. , M. Mastrototaro , A. Rossi , et al. 2009. “Aquaporin‐4 Orthogonal Arrays of Particles Are the Target for Neuromyelitis Optica Autoantibodies.” Glia 57, no. 13: 1363–1373. 10.1002/glia.20855. 19229993

[glia70024-bib-0077] Nielsen, S. , L. S. King , B. M. Christensen , and P. Agre . 1997b. “Aquaporins in Complex Tissues. II. Subcellular Distribution in Respiratory and Glandular Tissues of Rat.” American Journal of Physiology‐Cell Physiology 273, no. 5: C1549–C1561. 10.1152/ajpcell.1997.273.5.C1549. 9374640

[glia70024-bib-0078] Nielsen, S. , E. A. Nagelhus , M. Amiry‐Moghaddam , C. Bourque , P. Agre , and O. P. Ottersen . 1997a. “Specialized Membrane Domains for Water Transport in Glial Cells: High‐Resolution Immunogold Cytochemistry of Aquaporin‐4 in Rat Brain.” Journal of Neuroscience 17, no. 1: 171. 10.1523/JNEUROSCI.17-01-00171.1997. 8987746 PMC6793699

[glia70024-bib-0079] Niimura, Y. 2012. “Olfactory Receptor Multigene Family in Vertebrates: From the Viewpoint of Evolutionary Genomics.” Current Genomics 13, no. 2: 103–114. 10.2174/138920212799860706. 23024602 PMC3308321

[glia70024-bib-0080] Nomura, T. , S. Takahashi , and T. Ushiki . 2004. “Cytoarchitecture of the Normal Rat Olfactory Epithelium: Light and Scanning Electron Microscopic Studies.” Archives of Histology and Cytology 67, no. 2: 159–170. 10.1679/aohc.67.159. 15468955

[glia70024-bib-0081] Oshio, K. , D. K. Binder , B. Yang , S. Schecter , A. S. Verkman , and G. T. Manley . 2004. “Expression of Aquaporin Water Channels in Mouse Spinal Cord.” Neuroscience 127, no. 3: 685–693. 10.1016/j.neuroscience.2004.03.016. 15283967

[glia70024-bib-0082] Palazzo, C. , P. Abbrescia , O. Valente , et al. 2020. “Tissue Distribution of the Readthrough Isoform of AQP4 Reveals a Dual Role of AQP4ex Limited to CNS.” International Journal of Molecular Sciences 21, no. 4: 1531. 10.3390/ijms21041531. 32102323 PMC7073200

[glia70024-bib-0083] Palazzo, C. , C. Buccoliero , M. G. Mola , et al. 2019. “AQP4ex Is Crucial for the Anchoring of AQP4 at the Astrocyte End‐Feet and for Neuromyelitis Optica Antibody Binding.” Acta Neuropathologica Communications 7, no. 1: 51. 10.1186/s40478-019-0707-5. 30935410 PMC6444679

[glia70024-bib-0084] Papadopoulos, M. C. , G. T. Manley , S. Krishna , and A. S. Verkman . 2004. “Aquaporin‐4 Facilitates Reabsorption of Excess Fluid in Vasogenic Brain Edema.” FASEB Journal 18, no. 11: 1291–1293. 10.1096/fj.04-1723fje. 15208268

[glia70024-bib-0085] Parpura, V. , T. A. Basarsky , F. Liu , K. Jeftinija , S. Jeftinija , and P. G. Haydon . 1994. “Glutamate‐Mediated Astrocyte‐Neuron Signalling.” Nature 369, no. 6483: 744–747. 10.1038/369744a0. 7911978

[glia70024-bib-0086] Pastrana, E. , M. T. Moreno‐Flores , E. N. Gurzov , J. Avila , F. Wandosell , and J. Diaz‐Nido . 2006. “Genes Associated With Adult Axon Regeneration Promoted by Olfactory Ensheathing Cells: A New Role for Matrix Metalloproteinase 2.” Journal of Neuroscience 26, no. 20: 5347–5359. 10.1523/JNEUROSCI.1111-06.2006. 16707787 PMC6675307

[glia70024-bib-0087] Pati, R. , C. Palazzo , O. Valente , et al. 2022. “The Readthrough Isoform AQP4ex Is Constitutively Phosphorylated in the Perivascular Astrocyte Endfeet of Human Brain.” Biomolecules 12, no. 5: 633. 10.3390/biom12050633. 35625560 PMC9138620

[glia70024-bib-0088] Pietra, G. , M. Dibattista , A. Menini , J. Reisert , and A. Boccaccio . 2016. “The Ca2+‐Activated Cl‐ Channel TMEM16B Regulates Action Potential Firing and Axonal Targeting in Olfactory Sensory Neurons.” Journal of General Physiology 148, no. 4: 293–311. 10.1085/jgp.201611622. 27619419 PMC5037344

[glia70024-bib-0089] R Core Team . 2021. R: A Language and Environment for Statistical Computing. R Foundation for Statistical Computing.

[glia70024-bib-0090] Rash, J. E. , K. G. V. Davidson , N. Kamasawa , et al. 2005. “Ultrastructural Localization of Connexins (Cx36, Cx43, Cx45), Glutamate Receptors and Aquaporin‐4 in Rodent Olfactory Mucosa, Olfactory Nerve and Olfactory Bulb.” Journal of Neurocytology 34, no. 3: 307–341. 10.1007/s11068-005-8360-2. 16841170 PMC1525003

[glia70024-bib-0091] Rash, J. E. , T. Yasumura , C. S. Hudson , P. Agre , and S. Nielsen . 1998. “Direct Immunogold Labeling of Aquaporin‐4 in Square Arrays of Astrocyte and Ependymocyte Plasma Membranes in Rat Brain and Spinal Cord.” Proceedings of the National Academy of Sciences 95, no. 20: 11981–11986. 10.1073/pnas.95.20.11981. PMC21751 9751776

[glia70024-bib-0092] Rojas‐Mayorquín, A. E. , N. M. Torres‐Ruíz , D. Ortuño‐Sahagún , and G. Gudiño‐Cabrera . 2008. “Microarray Analysis of Striatal Embryonic Stem Cells Induced to Differentiate by Ensheathing Cell Conditioned Media.” Developmental Dynamics 237, no. 4: 979–994. 10.1002/dvdy.21489. 18351659

[glia70024-bib-0093] Rossi, A. , F. Pisani , G. P. Nicchia , M. Svelto , and A. Frigeri . 2010. “Evidences for a Leaky Scanning Mechanism for the Synthesis of the Shorter M23 Protein Isoform of Aquaporin‐4: Implication in Orthogonal Array Formation and Neuromyelitis Optica Antibody Interaction2.” Journal of Biological Chemistry 285, no. 7: 4562–4569. 10.1074/jbc.M109.069245. 20007705 PMC2836061

[glia70024-bib-0094] Rouach, N. , A. Koulakoff , V. Abudara , K. Willecke , and C. Giaume . 2008. “Astroglial Metabolic Networks Sustain Hippocampal Synaptic Transmission.” Science (New York, N.Y.) 322, no. 5907: 1551–1555. 10.1126/science.1164022. 19056987

[glia70024-bib-0096] Sakai, H. , K. Sato , Y. Kai , et al. 2014. “Distribution of Aquaporin Genes and Selection of Individual Reference Genes for Quantitative Real‐Time RT‐PCR Analysis in Multiple Tissues of the Mouse.” Canadian Journal of Physiology and Pharmacology 92, no. 9: 789–796. 10.1139/cjpp-2014-0157. 25188728

[glia70024-bib-0097] Scharfman, H. E. , and D. K. Binder . 2013. “Aquaporin‐4 Water Channels and Synaptic Plasticity in the Hippocampus.” Neurochemistry International 63, no. 7: 702–711. 10.1016/j.neuint.2013.05.003. 23684954 PMC3783552

[glia70024-bib-0098] Schindelin, J. , I. Arganda‐Carreras , E. Frise , et al. 2012. “Fiji: An Open‐Source Platform for Biological‐Image Analysis.” Nature Methods 9, no. 7: 676–682. 10.1038/nmeth.2019. 22743772 PMC3855844

[glia70024-bib-0099] Scott, J. W. , L. Sherrill , J. Jiang , and K. Zhao . 2014. “Tuning to Odor Solubility and Sorption Pattern in Olfactory Epithelial Responses.” Journal of Neuroscience 34, no. 6: 2025. 10.1523/JNEUROSCI.3736-13.2014. 24501345 PMC3913860

[glia70024-bib-0100] Silberstein, C. , R. Bouley , Y. Huang , et al. 2004. “Membrane Organization and Function of M1 and M23 Isoforms of Aquaporin‐4 in Epithelial Cells.” American Journal of Physiology. Renal Physiology 287, no. 3: F501–F511. 10.1152/ajprenal.00439.2003. 15149973

[glia70024-bib-0101] Skucas, V. A. , I. B. Mathews , J. Yang , et al. 2011. “Impairment of Select Forms of Spatial Memory and Neurotrophin‐Dependent Synaptic Plasticity by Deletion of Glial Aquaporin‐4.” Journal of Neuroscience 31, no. 17: 6392–6397. 10.1523/JNEUROSCI.6249-10.2011. 21525279 PMC3107562

[glia70024-bib-0102] Smith, K. E. , K. Whitcroft , S. Law , P. Andrews , D. Choi , and D. J. Jagger . 2020. “Olfactory Ensheathing Cells From the Nasal Mucosa and Olfactory Bulb Have Distinct Membrane Properties.” Journal of Neuroscience Research 98, no. 5: 888–901. 10.1002/jnr.24566. 31797433

[glia70024-bib-0103] Solbu, T. T. , and T. Holen . 2012. “Aquaporin Pathways and Mucin Secretion of Bowman's Glands Might Protect the Olfactory Mucosa.” Chemical Senses 37, no. 1: 35–46. 10.1093/chemse/bjr063. 21745799

[glia70024-bib-0104] Solenov, E. , H. Watanabe , G. T. Manley , and A. S. Verkman . 2004. “Sevenfold‐Reduced Osmotic Water Permeability in Primary Astrocyte Cultures From AQP‐4‐Deficient Mice, Measured by a Fluorescence Quenching Method.” American Journal of Physiology‐Cell Physiology 286, no. 2: C426–C432. 10.1152/ajpcell.00298.2003. 14576087

[glia70024-bib-0105] Strohschein, S. , K. Hüttmann , S. Gabriel , D. K. Binder , U. Heinemann , and C. Steinhäuser . 2011. “Impact of Aquaporin‐4 Channels on K+ Buffering and Gap Junction Coupling in the Hippocampus.” Glia 59, no. 6: 973–980. 10.1002/glia.21169. 21446052

[glia70024-bib-0106] Suzuki, Y. , M. Takeda , and A. I. Farbman . 1996. “Supporting Cells as Phagocytes in the Olfactory Epithelium After Bulbectomy.” Journal of Comparative Neurology 376, no. 4: 509–517. 10.1002/(SICI)1096-9861(19961223)376:4<509::AID-CNE1>3.0.CO;2-5. 8978466

[glia70024-bib-0107] Villar, P. S. , R. Delgado , C. Vergara , J. G. Reyes , and J. Bacigalupo . 2017. “Energy Requirements of Odor Transduction in the Chemosensory Cilia of Olfactory Sensory Neurons Rely on Oxidative Phosphorylation and Glycolytic Processing of Extracellular Glucose.” Journal of Neuroscience 37: 5736–5743. 10.1523/JNEUROSCI.2640-16.2017. 28500222 PMC6596473

[glia70024-bib-0108] Vogalis, F. , C. C. Hegg , and M. T. Lucero . 2005a. “Ionic Conductances in Sustentacular Cells of the Mouse Olfactory Epithelium.” Journal of Physiology 562, no. 3: 785–799. 10.1113/jphysiol.2004.079228. 15611020 PMC1665525

[glia70024-bib-0109] Vogalis, F. , C. C. Hegg , and M. T. Lucero . 2005b. “Electrical Coupling in Sustentacular Cells of the Mouse Olfactory Epithelium.” Journal of Neurophysiology 94, no. 2: 1001–1012. 10.1152/jn.01299.2004. 15788515

[glia70024-bib-0110] Wickham, H. 2016. ggplot2: Elegant Graphics for Data Analysis. Springer‐Verlag New York.

[glia70024-bib-0111] Woodley, S. K. , and M. J. Baum . 2003. “Effects of Sex Hormones and Gender on Attraction Thresholds for Volatile Anal Scent Gland Odors in Ferrets.” Hormones and Behavior 44, no. 2: 110–118. 10.1016/S0018-506X(03)00126-0. 13129482

[glia70024-bib-0112] Wrenn, C. C. , J. W. Kinney , L. K. Marriott , et al. 2004. “Learning and Memory Performance in Mice Lacking the GAL‐R1 Subtype of Galanin Receptor.” European Journal of Neuroscience 19, no. 5: 1384–1396. 10.1111/j.1460-9568.2004.03214.x. 15016096

[glia70024-bib-0113] Yang, M. , and J. N. Crawley . 2009. “Simple Behavioral Assessment of Mouse Olfaction.” Current Protocols in Neuroscience 48, no. 1: 8.24.1–8.24.12. 10.1002/0471142301.ns0824s48. PMC2753229 19575474

[glia70024-bib-0114] Zhang, L. , X. Zhuang , P. Kotitalo , et al. 2021. “Intravenous Transplantation of Olfactory Ensheathing Cells Reduces Neuroinflammation After Spinal Cord Injury via Interleukin‐1 Receptor Antagonist.” Theranostics 11, no. 3: 1147–1161. 10.7150/thno.52197. 33391526 PMC7738890

[glia70024-bib-0115] Zhao, H. , L. Ivic , J. M. Otaki , M. Hashimoto , K. Mikoshiba , and S. Firestein . 1998. “Functional Expression of a Mammalian Odorant Receptor.” Science 279, no. 5348: 237–242. 10.1126/science.279.5348.237. 9422698

